# Development of the Commercial Manufacturing Process for Nirmatrelvir in 17 Months

**DOI:** 10.1021/acscentsci.3c00145

**Published:** 2023-03-29

**Authors:** Christophe Allais, Christina G. Connor, Nga M. Do, Samir Kulkarni, Johnny W. Lee, Taegyo Lee, Emma McInturff, Jared Piper, Dave W. Place, John A. Ragan, R. Matt Weekly

**Affiliations:** Pfizer Worldwide Research & Development, Chemical Research & Development, Groton, Connecticut 06355, United States

## Abstract

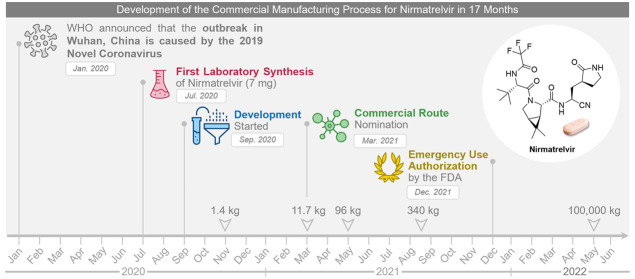

The advancement of nirmatrelvir, the active ingredient in Paxlovid, from discovery to emergency use authorization was achieved in just 17 months, requiring an unprecedented rate of chemical process development.

## INTRODUCTION

A novel coronavirus was identified in 2019, associated with a pneumonia outbreak in Wuhan, China, in December of that year.^1,2^ Sequencing studies of the virus (SARS-CoV-2) revealed a close homology with SARS-CoV-1, the virus that caused severe acute respiratory syndrome (SARS) in 2002.^3^ In March 2020 a special report summarizing publications and patents in the CAS content collection related to the disease was published in this journal.^4^ In response to these reports, Pfizer initiated a program to identify a small molecule, oral antiviral therapy based on inhibition of the 3CL protease involved in viral replication of SARS-CoV-2.

**Figure 1:**
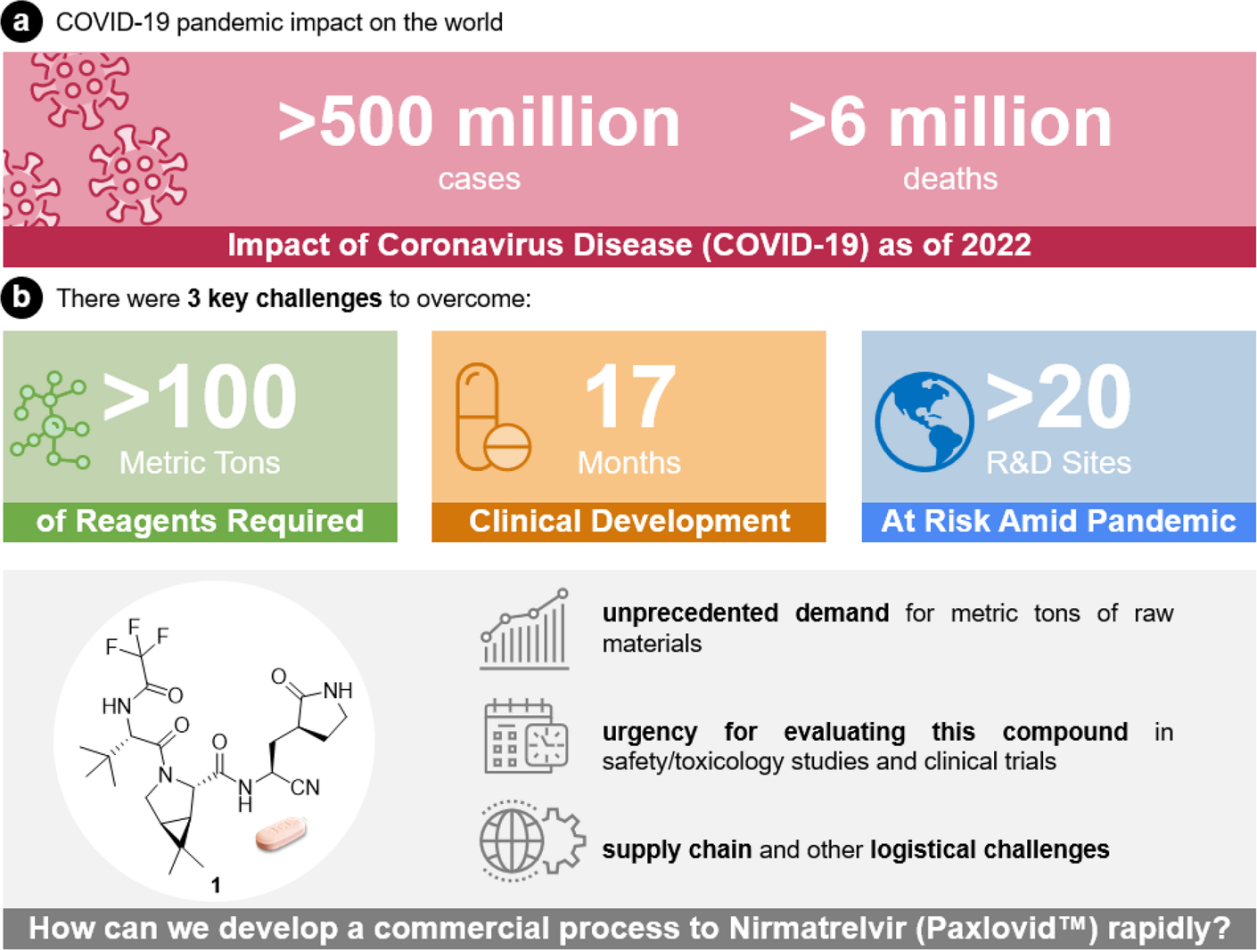
Overview of project challenges.

In November of 2021, Pfizer medicinal chemistry colleagues reported the discovery of PF-07321332 (nirmatrelvir), a potent, selective, and orally bioavailable inhibitor of SARS-CoV-2 M^pro^.^5,6^ The urgency for evaluating this compound in safety/toxicology studies and clinical trials, together with adoption of an aggressive, “lightspeed” development paradigm,^7^ led to an unprecedented demand for multikilogram quantities of this material. An overview of the project challenges is shown in Figure 1. The successful development and scale-up of an efficient chemical synthesis of nirmatrelvir allowed for progression of Paxlovid (the cotherapy of nirmatrelvir and ritonavir) from first laboratory synthesis (in July 2020) to FDA emergency use authorization^8^ (in December 2021), a period of just *17 months.* This is a new record for Pfizer and, to our knowledge, the pharmaceutical industry. This paper will provide a high-level overview of the synthetic chemistry, supply chain, and other logistical challenges that had to be addressed to achieve this goal.

**Figure 2:**
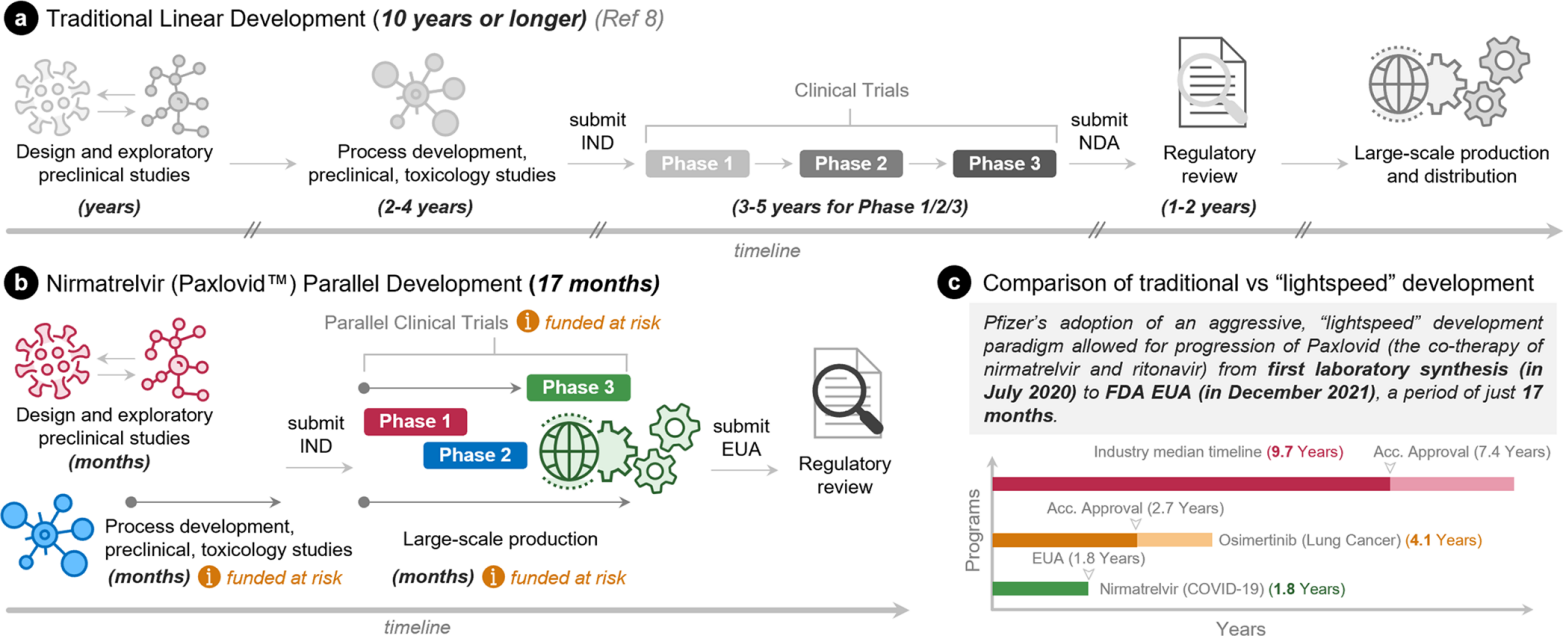
Development timeline comparisons.

Figure 2 shows project development timelines for a traditional, linear development paradigm compared to the more aggressive “lightspeed” development paradigm incorporated for Paxlovid,^7^ where “funded at risk” refers to the funding of these activities before the clinical safety and efficacy of the new therapy had been demonstrated. The industry median and osimertinib timelines in Figure 2c are from a recent review of clinical development timelines.^9^

### **Synthetic Strategy for Nirmatrelvir**

**Figure 3:**
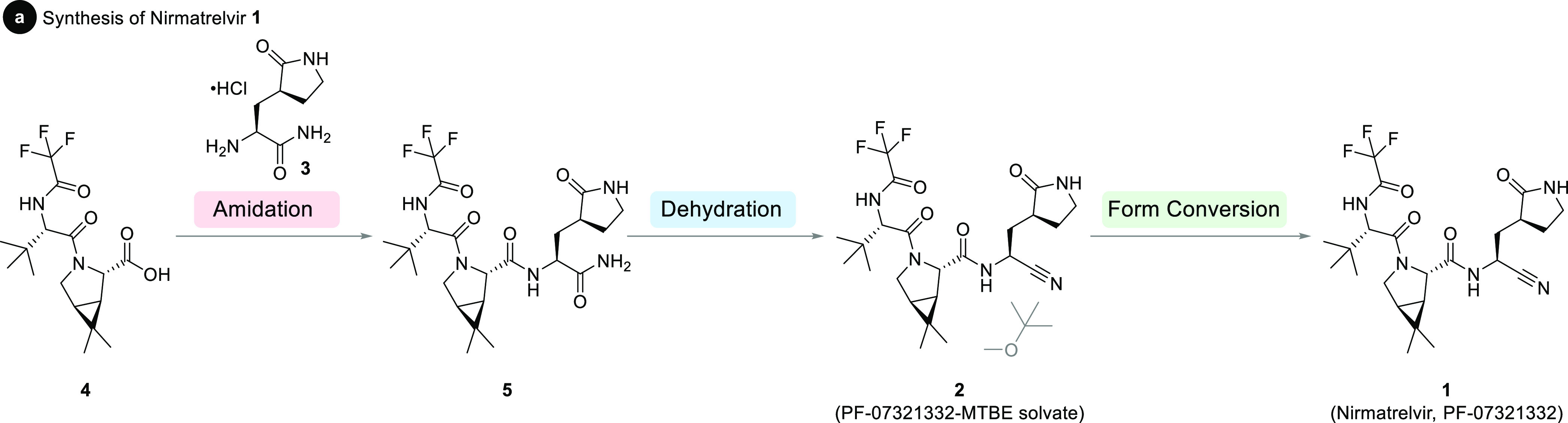
Final bond forming sequence to nirmatrelvir (**1**).

The synthetic strategy for preparation of nirmatrelvir used throughout development is shown in Figure 3. The end game is a three-step sequence: (i) amidation of primary amine **3** and acid **4** to form **5**; (ii) dehydration of the primary carboxamide **5** and isolation of the MTBE solvate (**2**); and (iii) form conversion to the anhydrous Form 1 of nirmatrelvir (**1**). The first cGMP (current Good Manufacturing Practice) campaigns, which supply material for regulatory toxicological and early clinical studies, followed this overall sequence, with variations of specific reagents, solvents, and final form isolations and recrystallizations to meet the necessary purity profiles based on the intended use of the final product. Maintaining common late-stage intermediates in this sequence (i.e., **5** to **2** to **1**) helped to keep a consistent purity profile in the final active pharmaceutical ingredient (API), an important consideration as the compound progressed through development and clinical studies so rapidly. Because of the peptidomimetic nature of the API, a series of amidation reactions was an obvious disconnection. Equally important was selection of starting materials and reagents that were readily available in large volumes (>100 t) and the syntheses of which utilized robust chemistry that allowed for ramping up production volumes in short timeframes.^10^ The complete synthesis is shown in Figure 11 and represents the process that was used at the time of commercial launch, including several improvements over the originally reported synthesis.^6^ Full details of the process development of the synthesis will be reported in future communications. The goal of this publication is to provide an overview of the development of this route and to describe how the accessibility of key building blocks allowed for an unprecedented rate of scale increase during development.

**Figure 4:**
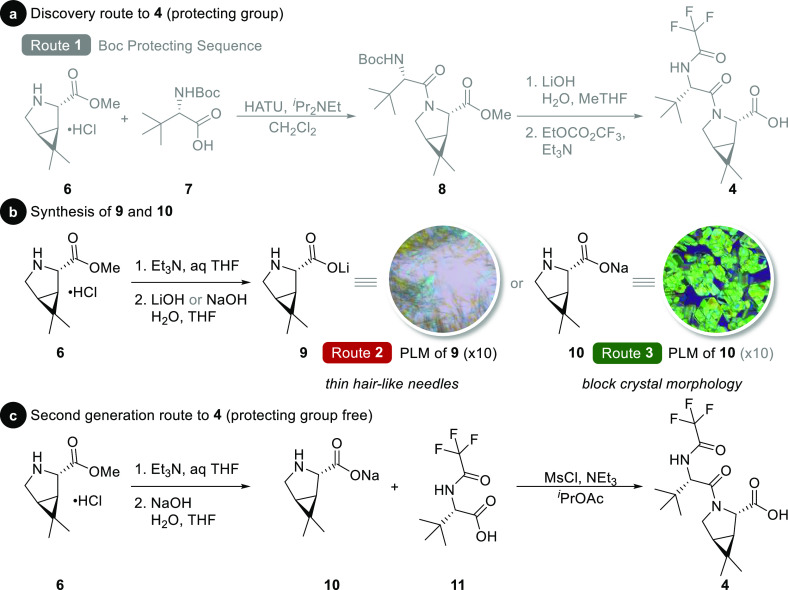
Synthesis of dipeptide **4**.

Three routes for the preparation of dipeptide **4** were utilized, as shown in Figure 4. Initially this intermediate was prepared by coupling of Boc-*tert*-leucine **7** and amine **6** followed by ester hydrolysis, Boc removal, and trifluoroacetylation (Figure 4a).^6^ A more convergent route that bypassed Boc-protection proceeded by ester saponification and isolation of the Li salt **9**, which was coupled with acid **11** to directly provide **4**. This route was successfully performed at scales exceeding 100 kg, during which several challenges were encountered with the physical properties of Li salt **9**, particularly the physical characteristics (thin, hairlike needles) that led to formation of thick slurries that would stagnate, separate, and form immobile superstructures in the reactor (Figure 4b). These slurries behaved like non-Newtonian fluids that suffered from strict process scale limitations and required specific vessel configurations and high agitation speeds to overcome the shear thinning properties and prevent immobilization. These properties hindered our ability to scale-up this chemistry across multiple manufacturing sites and led us to seek an alternate isolated intermediate that would avoid these problems. Several alternatives to the Li salt were evaluated including the HCl salt, zwitterion, and Na salt **10**. Although all three options showed potential advantages, the Na salt was the best positioned for rapid implementation. In addition to the salt switch, several changes were made to the step 2 amidation protocol to improve efficiency (Figure 4c). Full details of these optimization efforts will be reported in a future communication.

The Discovery route (Figure 4a) was used for the initial synthesis of nirmatrelvir and for scale-up to a 12 kg batch for support of regulatory toxicology and early clinical studies. The route with Li salt **9** was used to provide both clinical supplies (Phase 2 and 3) and API used under the emergency use authorization. The Na salt route (Figure 4c) is the commercial sequence and has also provided API for emergency use authorization. All three routes provide API that meets all quality specifications; the changes were driven by the need for greater efficiency and manufacturing logistics.

The timeline for introduction of the Na salt is representative of the remarkable development timeline that was realized with this program. The advancement from laboratory demonstration (2–5 g) through engineering and safety assessments (20–50 g), Kilo Lab campaign (2–5 kg), Pilot Plant campaign (20–50 kg), and transfer to commercial launch facility (200–1000 kg) would normally require 2–3 years, depending on the demand for clinical supplies and project prioritization. This timeline would include several stage gates, wherein internal R&D resources are balanced against the entire portfolio. Advancement through these various stage gates would require not only success in the clinic, but also favorable prioritization of the program relative to other development candidates. In the case of the “lightspeed” development protocol assigned to the Covid vaccine and oral protease inhibitor programs,^7^ these stage gate decision points were proceeded through with the assumption of success and the majority of development activities were prioritized and endorsed to be pursued at risk. In this context, “at risk” refers not to patient or worker safety, but rather to the financial and business risk of funding these activities before the clinical safety and efficacy of the new therapy had been demonstrated. Another example of financial commitments made at risk included purchase of starting materials at time points in advance of confirmation of clinical success and thus need for API supply. A benefit of this paradigm was that advancement from laboratory demonstration through successful scale-up to the commercial launch facility required just 13 weeks, a significant acceleration relative to the standard process, which is typically several quarters to over a year. This rate of development, requiring significant financial commitments prior to confirmed efficacy, is not viable for a standard development program. However, in the context of the global pandemic and urgent need for an effective oral therapy, the risks were warranted.^7^

### **Synthesis of Starting Materials**

**Figure 5:**
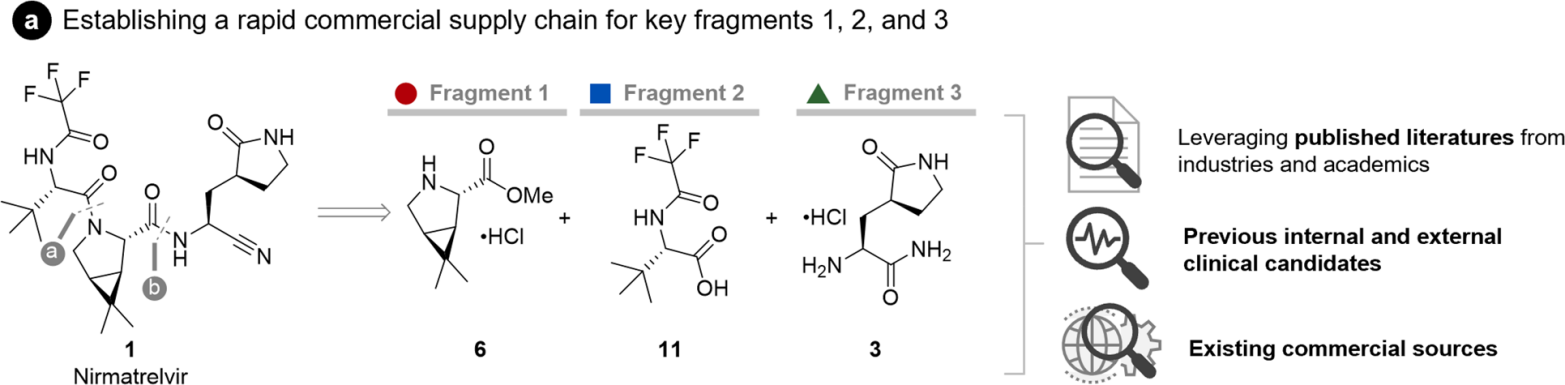
PF-07321332 building blocks.

The development routes and current synthesis (Figure 11) relied on three fundamental building blocks (Figure 5). The rapid scale increases successfully realized for nirmatrelvir required robust supply chains for each of these three starting materials. Fortunately, these supply chains could be rapidly established. This was the case for each of the three due to previous use in another pharmaceutical product (**6**), existing commercial sources (**11**), or leveraging a published synthesis that had been used for previous internal clinical candidates (**3**). This provides a vivid example of the importance of fundamental scientific research, and how work done for a project which was not commercialized^11^ can lay the groundwork for dramatic commercial breakthroughs in the future.

**Figure 6:**
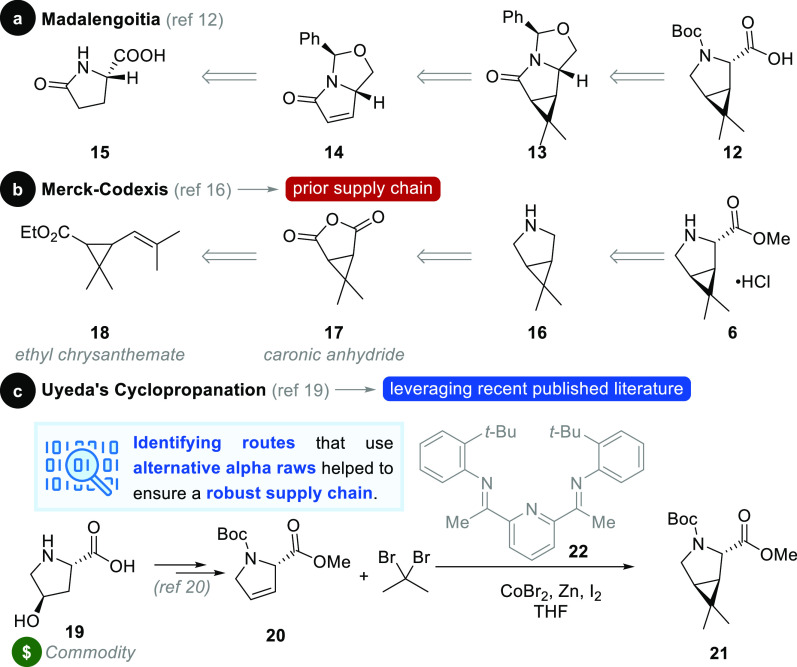
Synthetic routes to bicyclic pyrrolidine building block **6**.

The first starting material, bicyclic pyrrolidine **6**, can be prepared from Boc-protected bicyclic pyrrolidine acid **12**, which was first reported by Madalengoitia in 1999 in his studies of poly-l-proline type-II (PPII) secondary structure mimetics (Figure 6a).^12,13^ It was prepared from l-pyroglutamic acid via the tricyclic *N*,*O*-acetal **13**. Structurally similar to **12**, bicyclic pyrrolidine **6** was first reported in 2002^14^ and was a key component of the HCV protease inhibitor boceprevir.^15,16^ Codexis and Merck developed a monoamine oxidase (MAO)-based strategy^17^ for desymmetrization of the meso pyrrolidine **16** (Figure 6b), available in two steps from caronic anhydride (**17**), which in turn is available in two steps from ethyl chrysanthemate (**18**).^18^ Although boceprevir was withdrawn from the market in 2015, the existence of this enzymatic route allowed us to reestablish these supply chains rapidly and access the large quantities of amine **6** necessary for nirmatrelvir manufacture. Several other reported routes to **6** also proceed via caronic anhydride and thus rely on the same alpha raw.^18,19^ Identifying routes that use alternative alpha raws helped to ensure a robust supply chain. For example, application of Uyeda’s cobalt-catalyzed cyclopropanation^20^ of olefin **20**,^6^ which is available from hydroxyproline **19**,^21^ provided access to **6** without the need for ethyl chrysanthemate. The Uyeda cyclopropanation route (Figure 6c) has been demonstrated on commercial scale (200 kg batch size, >10 MT material produced) to provide starting material **6** that meets all purchase specifications and is thus available if needed should the supply chain to meso pyrrolidine **16** be compromised.

**Figure 7:**
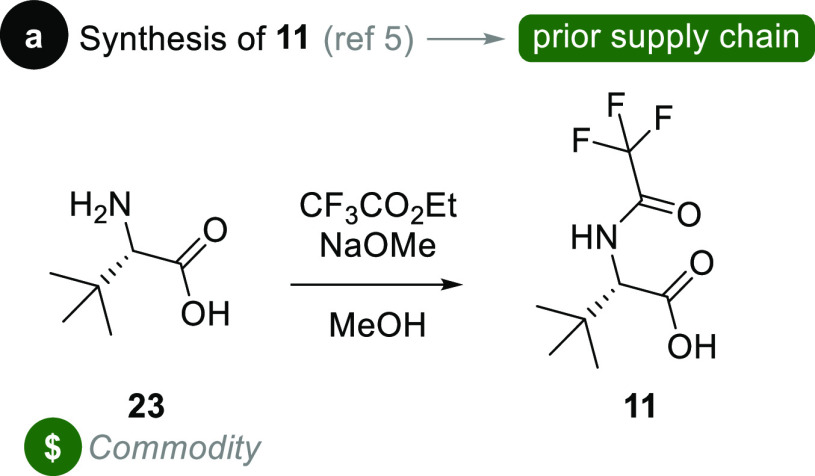
Synthesis of trifluoroacetamide **11**.

The second starting material, tertiary-leucine (also referred to as pseudoleucine) was first reported nearly 100 years ago.^22^ Tertiary leucine is commercially available, and conversion to the trifluoroacetamide **11** can be achieved by reaction with ethyl trifluoroacetate and sodium methoxide (see Figure 7).^6^

**Figure 8:**
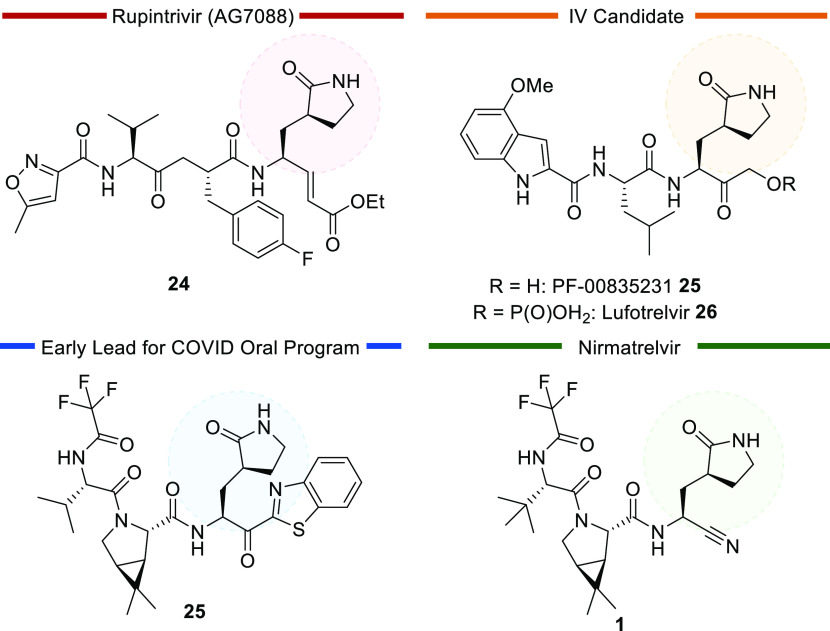
Protease inhibitors containing the chiral lactam moiety.

The third starting material is primary amide **3**, available from protected amino acid **30**. Boc ester **30** was first reported by Tian, Nayyar, and co-workers at Pfizer La Jolla in 2001, and was a key intermediate in the candidate rupintrivir (AG7088, **24**, Figure 8), a rhinovirus protease inhibitor.^23,24^ It was a key component of several subsequent protease inhibitors developed at Pfizer, including hydroxy ketone **25**, which was discovered in 2003 in a program directed at developing therapeutics against SARS CoV-1.^11^ It was also a key design element in an early lead for the Covid oral program (**25**) and in nirmatrelvir (**1**).^25^

**Figure 9:**
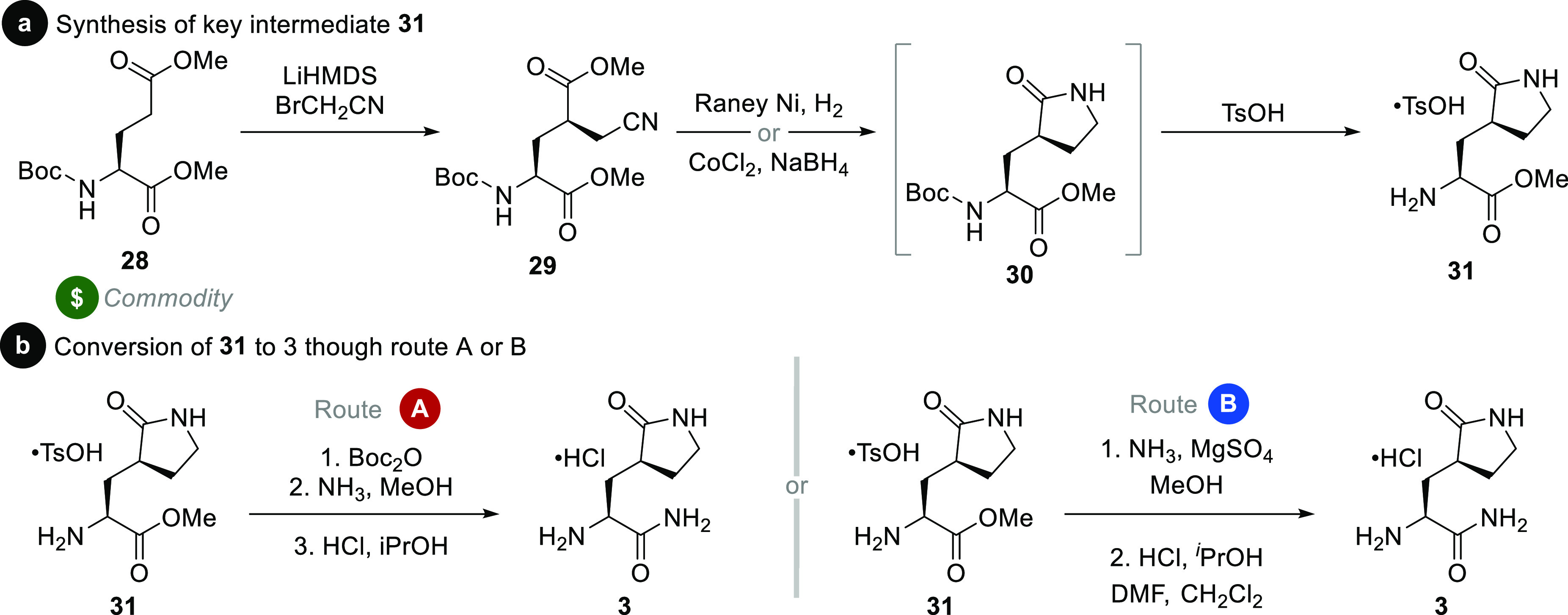
(a) Lithium dianion alkylation to control lactam stereochemistry. (b) Synthesis of carboxamide **3**.

The synthesis of **30** relies on a highly diastereoselective alkylation of the lithium dianion of Boc-dimethylglutarate as reported by Hanessian (Figure 9).^26^ Alkylation with bromoacetonitrile leads to nitrile **29** as predominantly one diastereomer. Nitrile reduction and cyclization forms lactam **30**. Although this synthetic route had not been previously utilized in a commercial product, the published synthesis provided a foundation for rapid tech transfer to multiple commercial suppliers, who were able to scale-up this chemistry and meet the aggressive bulk delivery timelines required for the successful development of nirmatrelvir. Importantly, another Pfizer team was focused on the IV candidate lufotrelvir (**26**), which is the phosphate pro-drug of PF-00835231 (**25**). Their work included significant optimization of the sequence shown in Figure 9a, including identification of tosylate salt **31** as a crystalline intermediate that avoided the need for chromatographic purification of intermediate **30**.^27^ Consequently, the nirmatrelvir team was able to leverage the availability of tosylate salt **31** in the preparation of carboxamide starting material **3**. The preparation of primary amide **3** is shown in Figure 9b. Two different routes were utilized to convert the methyl ester to carboxamide, reflecting preferences at different manufacturers. The first route proceeded via the Boc-protected amine (route A), while the other was a direct aminolysis of the tosylate salt followed by conversion to the HCl salt (route B).

**Figure 10:**
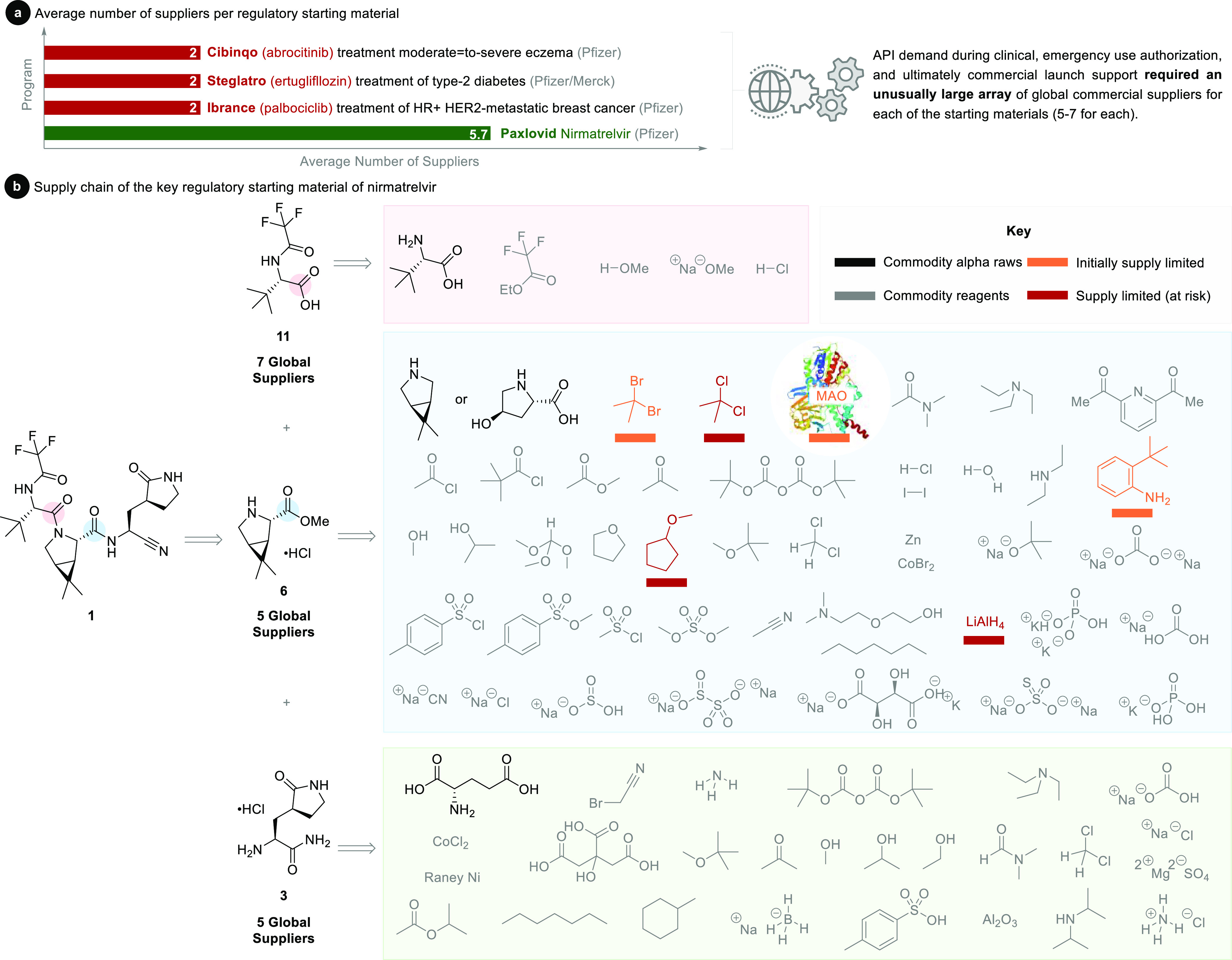
Supply chain for starting materials **3**, **6**, and **11**.

The supply chain for each of these materials is summarized in Figure 10. The complexity of the supply chain is reflected in the number of reagents and solvents required for execution of this chemistry at multiple vendors. For example, over 70 unique raw materials are utilized in the various supply nodes preparing the three starting materials. The significant API demand during clinical, emergency use authorization, and ultimately commercial launch support required an unusually large array of global commercial suppliers for each of the starting materials (5–7 for each). By way of contrast, for a standard new product, two suppliers would be in place for each starting material at time of filing, possibly with a third supplier identified as contingency. Expansion to a wider array of suppliers would occur postlaunch if commercial demand required it. The color shading in Figure 10 denotes commodity alpha raws (black) and reagents (gray), and materials that were supply limited initially (orange) or supply limited such that alternatives were required (red).

**Figure 11:**
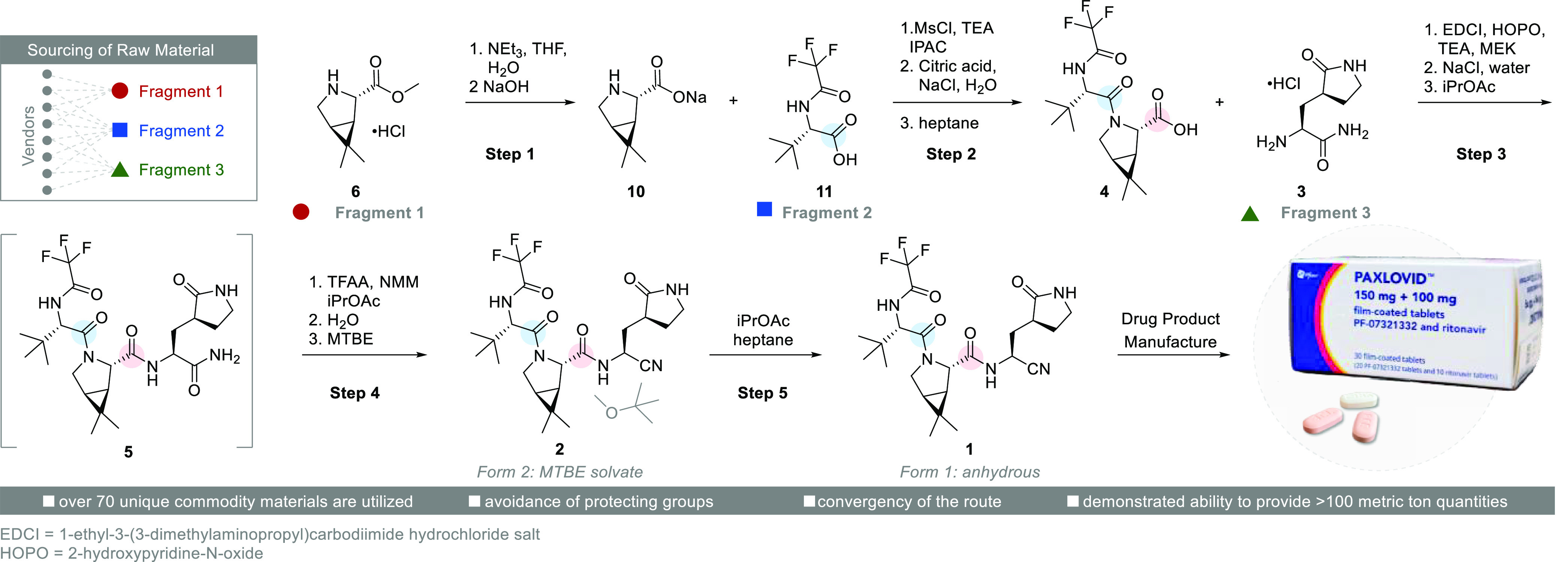
Current synthesis of nirmatrelvir.

The current synthesis of nirmatrelvir is shown in Figure 11. It comprises four chemical transformations, four isolations, and one form conversion. Alternative syntheses of nirmatrelvir have been reported by several groups.^28−30^ The route described in this work compares favorably in terms of convergency, absence of protecting groups, and demonstrated ability to provide >100 t quantities of nirmatrelvir.

Additional convergency can be realized by forming the nitrile prior to amide bond coupling, as demonstrated in the Lipshutz synthesis.^28^ We had investigated this strategy^6^ but found that detectable levels of cyanide were formed in reactions with aminonitriles (e.g., compound **3** with a nitrile replacing the primary carboxamide). Developing robust methods to address the risk of cyanide generation in large-scale manufacturing equipment could restrict our ability to meet the aggressive supply timelines of this program, so we instead pursued the strategy of dehydrating a primary nitrile after acylation of the amine to avoid this risk.

Reduction of waste and decreases in energy utilization and overall carbon footprint are improvements typically observed in a standard project development life cycle. Recognizing the importance of sustainability as one of the major and perhaps most exciting challenges pharmaceutical companies will face in decades to come, these parameters were also optimized despite the “lightspeed” aspect of the program. Process efficiency was improved significantly during development, employing a large team of process development scientists and engineers, with an aim to decrease the environmental impact of the manufacture of nirmatrelvir and improve throughput to supply a potential oral therapy for a global pandemic. This is demonstrated by the green chemistry metrics, shown in Table 1, which compare the initial process for clinical supply manufacture^6^ to the manufacturing route described in Figure 11.

**Table 1:**
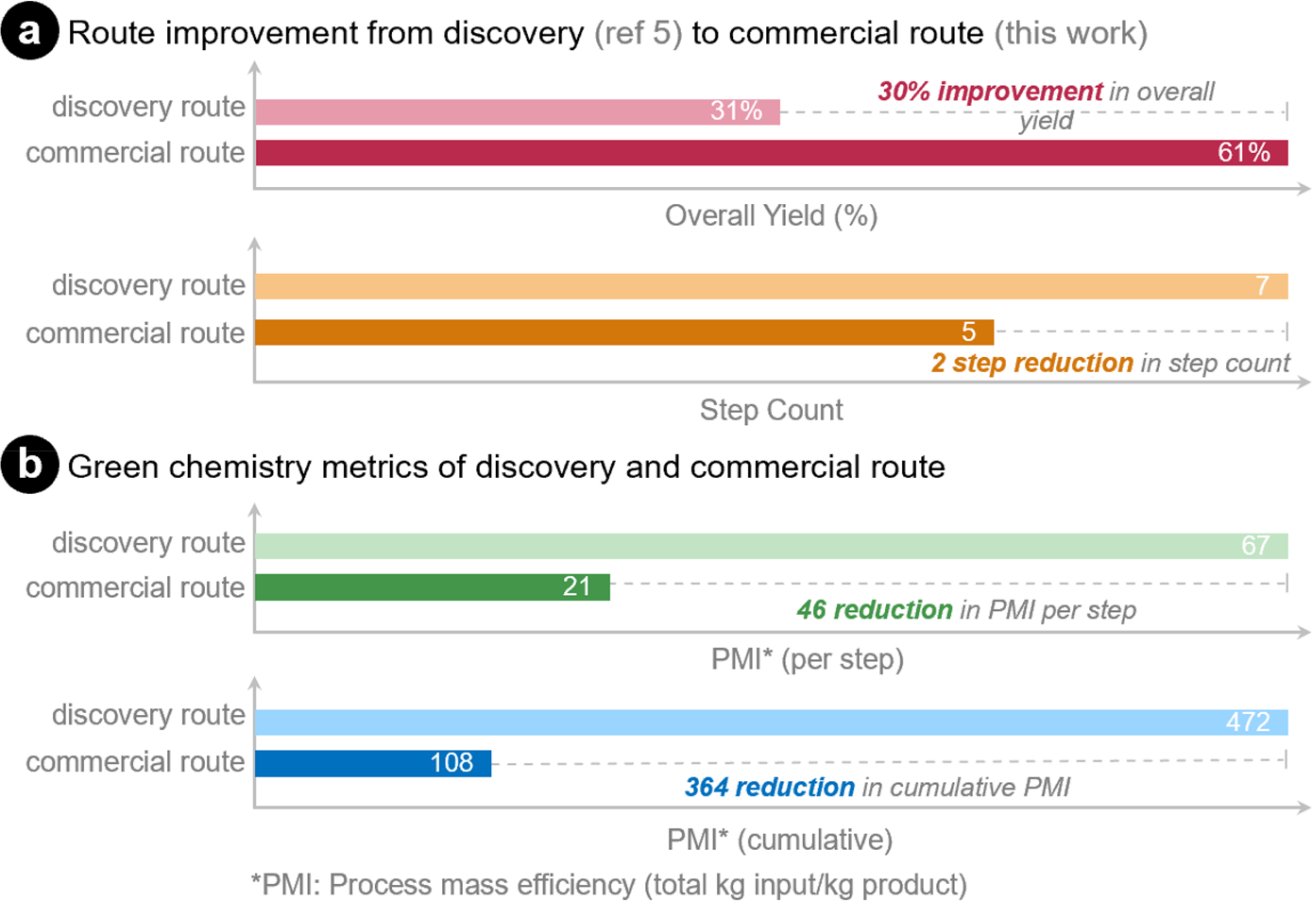
Green Chemistry Metrics for Initial Clinical Supply Route vs Current Route

## CONCLUSION

The synthesis of nirmatrelvir shown in Figure 11 has to date provided hundreds of metric tons of the API across multiple vendor sites. Further details of the experiments leading to the optimization of this five-step sequence will be reported in future manuscripts. The rapid development of the complex supply chains necessary for this project demonstrates the importance of previous research and how work done for earlier projects, whether commercialized^15,16^ or not,^11^ can lay the groundwork for dramatic future commercial breakthroughs. It also demonstrates the rapid timelines that can be realized when the urgency of an unmet medical need allows R&D investments to be made at-risk and without prioritization against other development projects.^7^

## Supporting Information Available

Experimental procedures and compound characterization for the reactions shown in Figures 11, 9b, and 6c (PDF)
