# Supplementary material for: Development of the Commercial Manufacturing Process for Nirmatrelvir in 17 Months
Source: ACS Cent Sci. 2023 Mar 29;9(5):849–57. doi: 10.1021/acscentsci.3c00145 (PMC10069651; doi:10.1021/acscentsci.3c00145)

Supplementary Information, 23 pages

# Development of the Commercial Manufacturing Process for Nirmatrelvir in 17 Months

Christophe Allais, Christina G. Connor, Nga M. Do, Samir Kulkarni, Johnny W. Lee, Taegyo Lee, Emma McInturff, Jared Piper, Dave W. Place, John A. Ragan,* R. Matt Weekly

Pfizer Worldwide Research & Development, Chemical Research & Development, Groton, CT 06355

*Corresponding author. Email: john.a.ragan@pfizer.com

Christophe.allais@pfizer.com

Christina.connor@pfizer.com

Nga.m.do@pfizer.com

Samir.kulkarni@pfizer.com

Johnny.lee@pfizer.com

Taegyo.lee@pfizer.com

Emma.mcinturff@pfizer.com

Jared.piper@pfizer.com

Dave.place@pfizer.com

Rodney.m.weekly@pfizer.com

## Experimental Procedures

### General

All reactions were carried out using continuous stirring under an atmosphere of nitrogen or argon gas unless otherwise noted. When appropriate, reaction vessels were dried under dynamic vacuum using a heat gun, and anhydrous solvents (Sure-Seal™ products from Aldrich Chemical Company, Milwaukee, Wisconsin or DriSolv™ products from EMD Chemicals, Gibbstown, NJ) were employed. Other commercial solvents and reagents were used without further purification.

Reaction progress was monitored using thin-layer chromatography (TLC), liquid chromatography-mass spectrometry (LCMS), high-performance liquid chromatography (HPLC), and/or gas chromatography-mass spectrometry (GCMS) analyses. TLC was

performed on pre-coated silica gel plates with a fluorescence indicator (254 nm excitation wavelength) and visualized under UV light and/or with $I_2$, $KMnO_4$, $CoCl_2$, phosphomolybdic acid, and/or ceric ammonium molybdate stains. LCMS data was acquired on an Agilent 1100 Series instrument with a Leap Technologies autosampler, Gemini C18 columns, acetonitrile/water gradients, and either trifluoroacetic acid, formic acid, or ammonium hydroxide modifiers. The column eluate was analyzed using a Waters ZQ mass spectrometer scanning in both positive and negative ion modes from 100 to 1200 Da. HPLC data were generally acquired on an Agilent 1100 Series instrument, using the columns indicated, acetonitrile/water gradients, and either trifluoroacetic acid or ammonium hydroxide modifiers. GCMS data were acquired using a Hewlett Packard 6890 oven with an HP 6890 injector, HP-1 column (12 m x 0.2 mm x 0.33 µm), and helium carrier gas. Samples were analyzed on an HP 5973 mass selective detector scanning from 50 to 550 Da using electron ionization.

Mass spectrometry data are reported from LCMS analyses. Mass spectrometry (MS) was performed via atmospheric pressure chemical ionization (APCI), electrospray ionization (ESI), electron impact ionization (EI) or electron scatter ionization (ES) sources. Proton nuclear magnetic spectroscopy ($^1$H NMR) chemical shifts are given in parts per million downfield from tetramethylsilane and were recorded on 300, 400, 500, or 600 MHz Varian, Bruker, or Jeol spectrometers. Chemical shifts are expressed in parts per million (ppm, $\delta$) referenced to the deuterated solvent residual peaks (chloroform, 7.26 ppm; $CD_2HOD$, 3.31 ppm; acetonitrile-$d_2$, 1.94 ppm; dimethyl sulfoxide-$d_5$, 2.50 ppm; DHO, 4.79 ppm). The peak shapes are described as follows: s, singlet; d, doublet; dd, doublet of doublet; ddd, doublet of doublet of doublet; dt, doublet of triplet; t, triplet; q, quartet; qd, quartet of doublet; quin, quintet; m, multiplet; br s, broad singlet; app, apparent. Analytical SFC data were generally acquired on a Berger analytical instrument as described above. Optical rotation data were acquired on a PerkinElmer model 343 polarimeter using a 1 dm cell. Microanalyses were performed by Quantitative Technologies Inc. and were within 0.4% of the calculated values.

Unless otherwise noted, chemical reactions were performed at room temperature (about 23 degrees Celsius). Unless noted otherwise, all reactants were obtained

commercially and used without further purification or were prepared using methods known in the literature.

The terms “concentrated”, “evaporated”, and “concentrated *in vacuo*” refer to the removal of solvent at reduced pressure on a rotary evaporator with a bath temperature less than 60 °C, or at a temperature as specified. The abbreviations “min” and “h” stand for “minutes” and “hours,” respectively. The term “TLC” refers to thin-layer chromatography, “room temperature or ambient temperature” means a temperature between 18 to 25 °C, “GCMS” refers to gas chromatography–mass spectrometry, “LCMS” refers to liquid chromatography–mass spectrometry, “UPLC” refers to ultra-performance liquid chromatography, “HPLC” refers to high-performance liquid chromatography, and “SFC” refers to supercritical fluid chromatography. Other abbreviations used include “°C” is degrees Celsius; “$CO_2$” is carbon dioxide; “eq.” or “equiv.” is equivalents; “DMSO-$d_6$” is hexadeutero dimethylsulfoxide; “g” is gram; “HCl” is hydrogen chloride; “HOPO” is 2-Hydroxypyridin-N-oxide; “HRMS” is high resolution mass spectroscopy; “Hz” is hertz; “iPrOAc” is isopropyl acetate; “K” is Kelvin; “kg” is kilogram; “L” is liter; “M” is mole or molar; “mbar” is millibar; “MEK” is methyl ethyl ketone; “MeOH” is methanol; “MHz” is megahertz; “mg” is milligrams; “μg” is micrograms; “min” is minutes; “mL” is milliliter; “μL” is microliter; “mm” is millimeter; “mmol” is millimole; “μmol” is micromole; “MTBE” is methyl tert-butyl ether; “NaCl” is sodium chloride; “$NaHCO_3$” is sodium bicarbonate; “$Na_2SO_4$” is sodium sulfate; “PXRD” is powder x-ray diffraction; and “THF” is tetrahydrofuran.

The following experimental procedures are either laboratory scale or small manufacturing scale (e.g. kilo lab) reactions which are representative of the procedures developed to support larger scale (50-200 kg) commercial manufacturing batches. Spectroscopic techniques including, $^1$H NMR, $^{13}$C NMR, IR and mass spec were utilized for initial characterization. During manufacturing scale-up, validated UPLC methods were developed for each step of the synthesis from compound **6** to nirmatrelvir (**1**).

Step 1: Preparation of sodium (1*R*,2*S*,5*S*)-6,6-dimethyl-3-azabicyclo[3.1.0]hexane-2-carboxylate (**10**)

*Step 1*

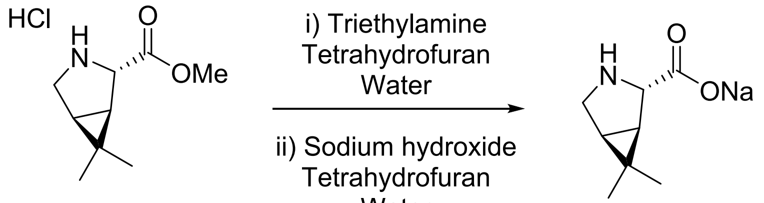


Methyl (1*R*,2*S*,5*S*)-6,6-dimethyl-3-azabicyclo[3.1.0]hexane-2-carboxylate, hydrochloride salt (compound **6**, 40 g, 195 mmol, 1.0 equivalents), tetrahydrofuran (80 mL, 2 mL/g of compound **6**) and water (40 mL, 1 mL/g of compound **6**) are combined and stirred at 25 °C. Triethylamine (40.7 mL, 292 mmol, 1.5 equivalents) is charged and the mixture is stirred for 30 minutes. The pH of the agitated mixture should be not less than 8.5. Agitation is stopped and the phases allowed to separate. The aqueous phase is removed to provide an organic solution of methyl (1*R*,2*S*,5*S*)-6,6-dimethyl-3-azabicyclo[3.1.0]hexane-2-carboxylate. In a separate vessel, sodium hydroxide (8.16 g, 204 mmol, 1.05 equivalents), tetrahydrofuran (360 mL, 9 mL/g of compound **6**) and water (40 mL, 1 mL/g of compound **6**) are combined and heated to 40 °C with stirring. The organic solution of methyl (1*R*,2*S*,5*S*)-6,6-dimethyl-3-azabicyclo[3.1.0]hexane-2-carboxylate is added to this mixture over not less than 15 minutes, and the resulting mixture is stirred for 4 hours at 40 °C. A sample is analyzed for reaction completion by UPLC (target of not more than 4% methyl (1*R*,2*S*,5*S*)-6,6-dimethyl-3-azabicyclo[3.1.0]hexane-2-carboxylate remaining, continue stirring if the reaction is not complete). Upon completion, the mixture is cooled to 20 °C and stirred for not less than 2 hours. The solids are collected by filtration, rinsed with 96:4 THF/water (80 mL), and dried at 70 °C in a vacuum oven to provide sodium (1*R*,2*S*,5*S*)-6,6-dimethyl-3-azabicyclo[3.1.0]hexane-2-carboxylate, compound **10**.

$^{1}$H NMR (600 MHz, $CD_3OD$-$d_4$, 298K): $\delta$ 3.37 (d, 1H), 3.31 (dd, 1H), 2.76 (dd, 1H), 1.62 (dd, 1H), 1.36 (m, 1H), 1.06 (s, 3H), 1.03 (s, 3H).

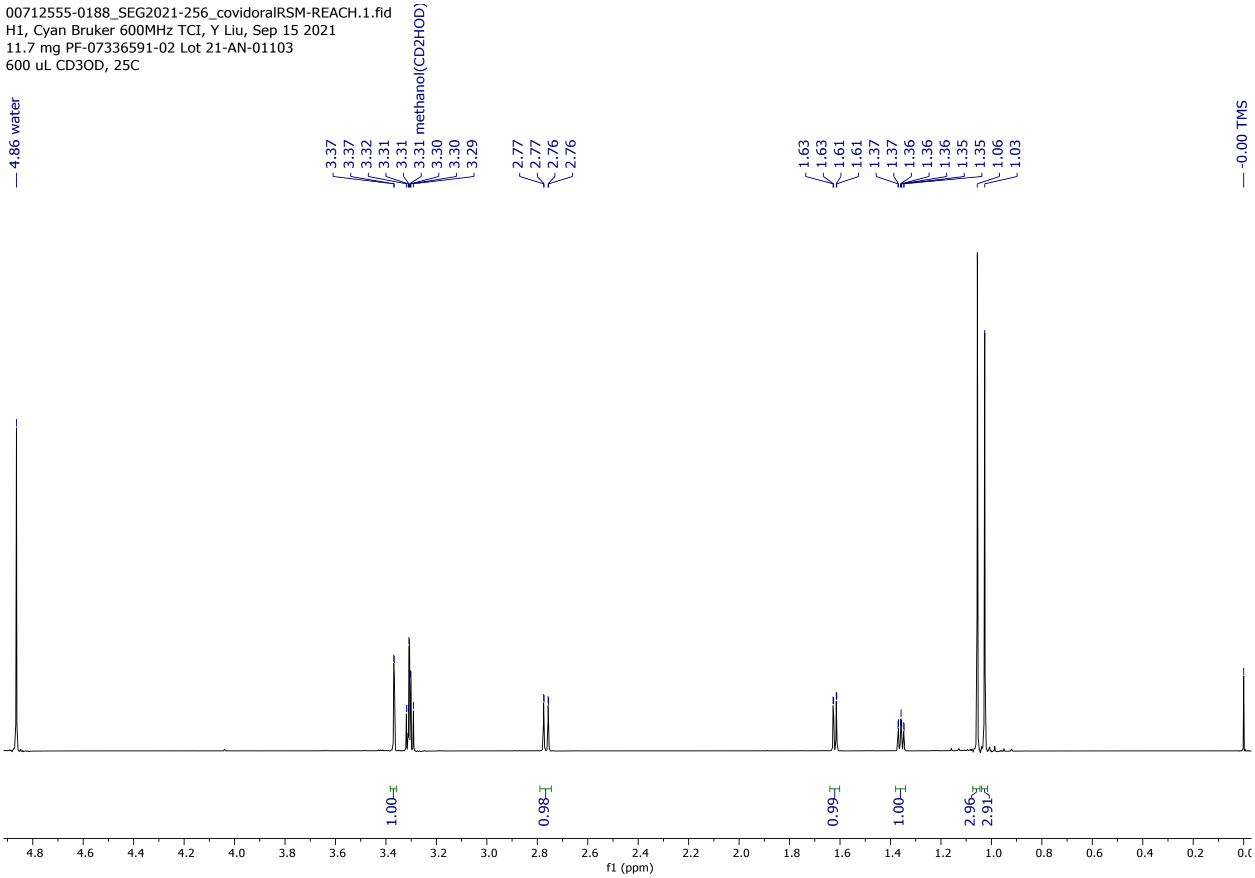


$^{13}$C NMR (150 MHz, $CD_3OD$-$d_4$, 298K): δ 181.6, 64.3, 47.2, 37.7, 31.6, 27.1, 20.9, 14.2.

HRMS: (ESI$^+$) Calcd for $C_8H_{14}O_2N^+$: 156.1019, Found: 156.1020 (mass deviation +0.83 ppm)

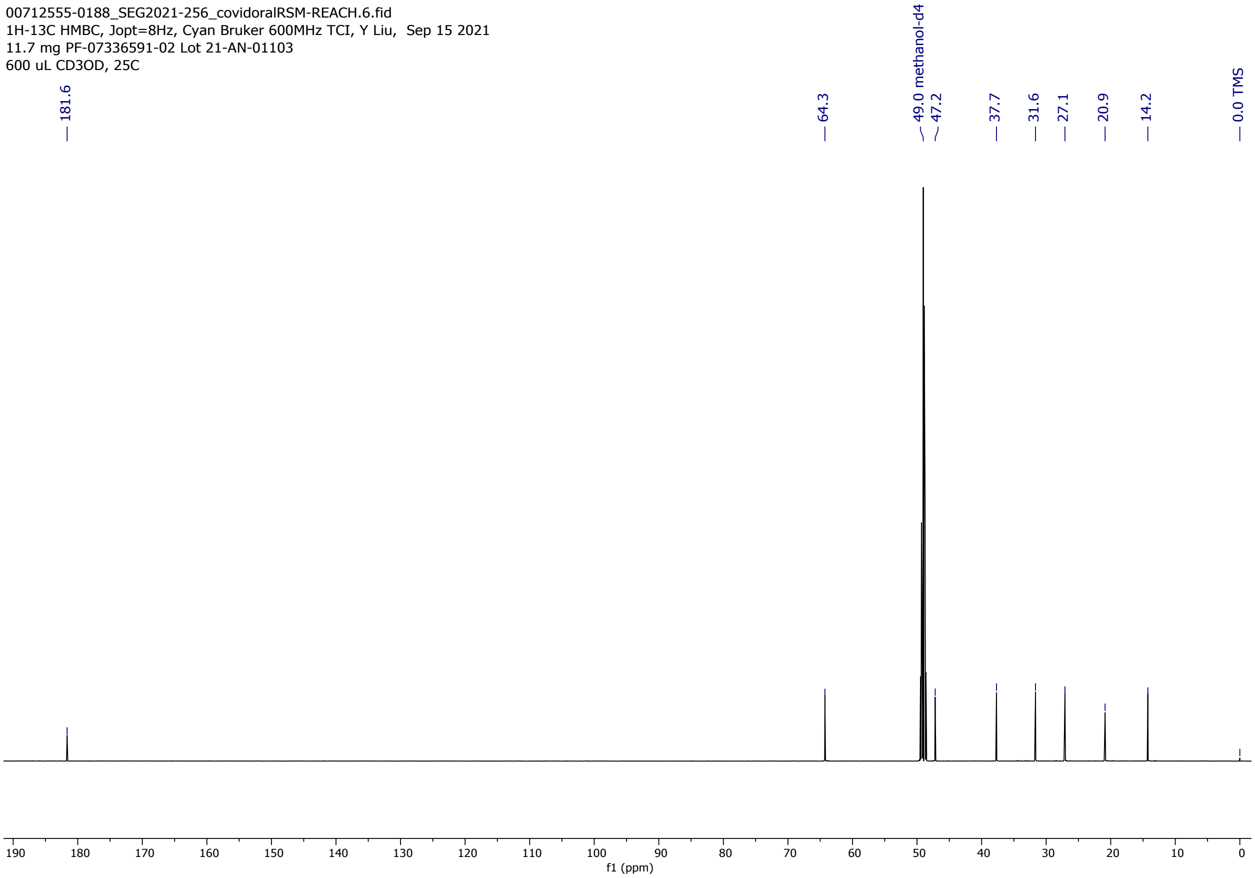


Step 2: Preparation of (1*R*,2*S*,5*S*)-3-((S)-3,3-dimethyl-2-(2,2,2-trifluoroacetamido) butanoyl)-6,6-dimethyl-3-azabicyclo[3.1.0]hexane-2-carboxylic acid, compound **4**

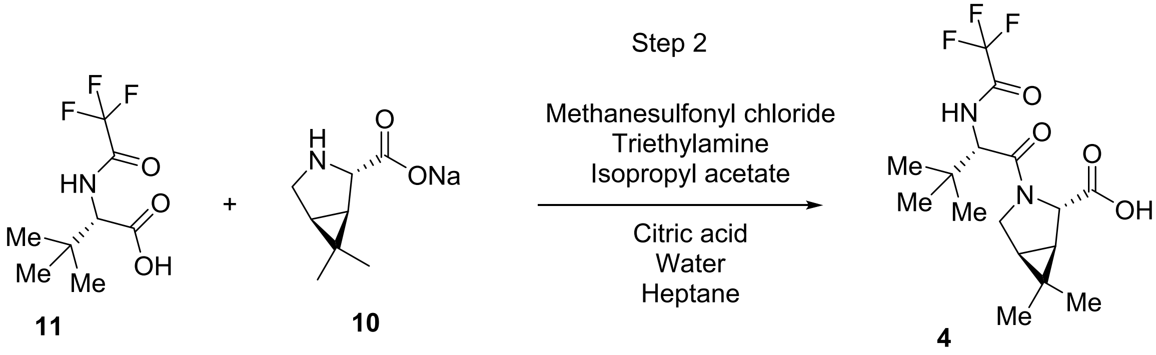


(*S*)-3,3-dimethyl-2-(2,2,2-trifluoroacetamido)butanoic acid, compound **11** (38.9 g, 169 mmol, 1.2 equivalents), methanesulfonyl chloride (17.8 g, 155 mmol, 1.1 equivalents) and isopropyl acetate (500 mL, 20 mL/g of compound **10**) are combined and stirred at 20 °C. Triethylamine (49.0 mL, 423 mmol, 2.5 equivalents) is charged at a rate such that the

reaction temperature does not exceed 25 °C, and the resulting mixture is stirred for 1 hour. Sodium (1*R*,2*S*,5*S*)-6,6-dimethyl-3-azabicyclo[3.1.0] hexane-2-carboxylate, compound **10** (25.0 g, 141 mmol, 1.0 equivalents) is charged, and the mixture stirred for 4 hours. A sample of the reaction mixture is obtained and analyzed for reaction completion (not more than 3% **10** by UPLC). If reaction is not complete, additional triethylamine may be added. The reaction mixture is quenched by addition of aqueous citric acid (74 g citric acid monohydrate, 353 mmol, 2.5 equivalents, in 150 mL water), and the mixture heated to 40 °C. The mixture is stirred for at least 10 minutes, then the layers are allowed to settle. The aqueous phase is removed, and the organic phase is washed with 2 portions of water (125 mL each). The organic phase is cooled to 10-15 °C and concentrated by vacuum distillation (-100 mbar, gradually warming to a maximum jacket temperature of 60 °C) to a volume of approximately 192 mL. The mixture is analyzed for water content (Karl-Fischer); if greater than 3 wt% water, the vacuum distillation is repeated with additional isopropyl acetate. The solution is heated to 60 °C and heptane (192 mL) is added. The mixture is stirred and cooled to 10 °C over approximately 4 hours. The slurry is stirred at 10 °C for 3 hours. Solids are collected by filtration and rinsed with 1:1 iPrOAc/heptane (100 mL). The solids are dried at 50 °C in a vacuum oven to provide (1*R*,2*S*,5*S*)-3-((S)-3,3-dimethyl-2-(2,2,2-trifluoroacetamido)butanoyl)-6,6-dimethyl-3-azabicyclo[3.1.0]hexane-2-carboxylic acid, compound **4**.

Note: in the $^{1}H$ and $^{13}C$ NMR spectra, 2 sets of resonances were observed due to the presence of both *E* and *Z* amide bond rotamers in solution. Only the major resonances (*Z* rotamer, 92%) are listed here.

$^{1}H$ NMR (600 MHz, DMSO-$d_6$, 298K): $\delta$ 12.72 (s, 1H), 9.42 (d, 1H), 4.44 (d, 1H), 4.15 (s, 1H), 3.85 (dd, 1H), 3.72 (d, 1H), 1.53 (dd, 1H), 1.41 (d, 1H), 1.01 (s, 3H), 1.00 (s, 9H), 0.82 (s, 3H).

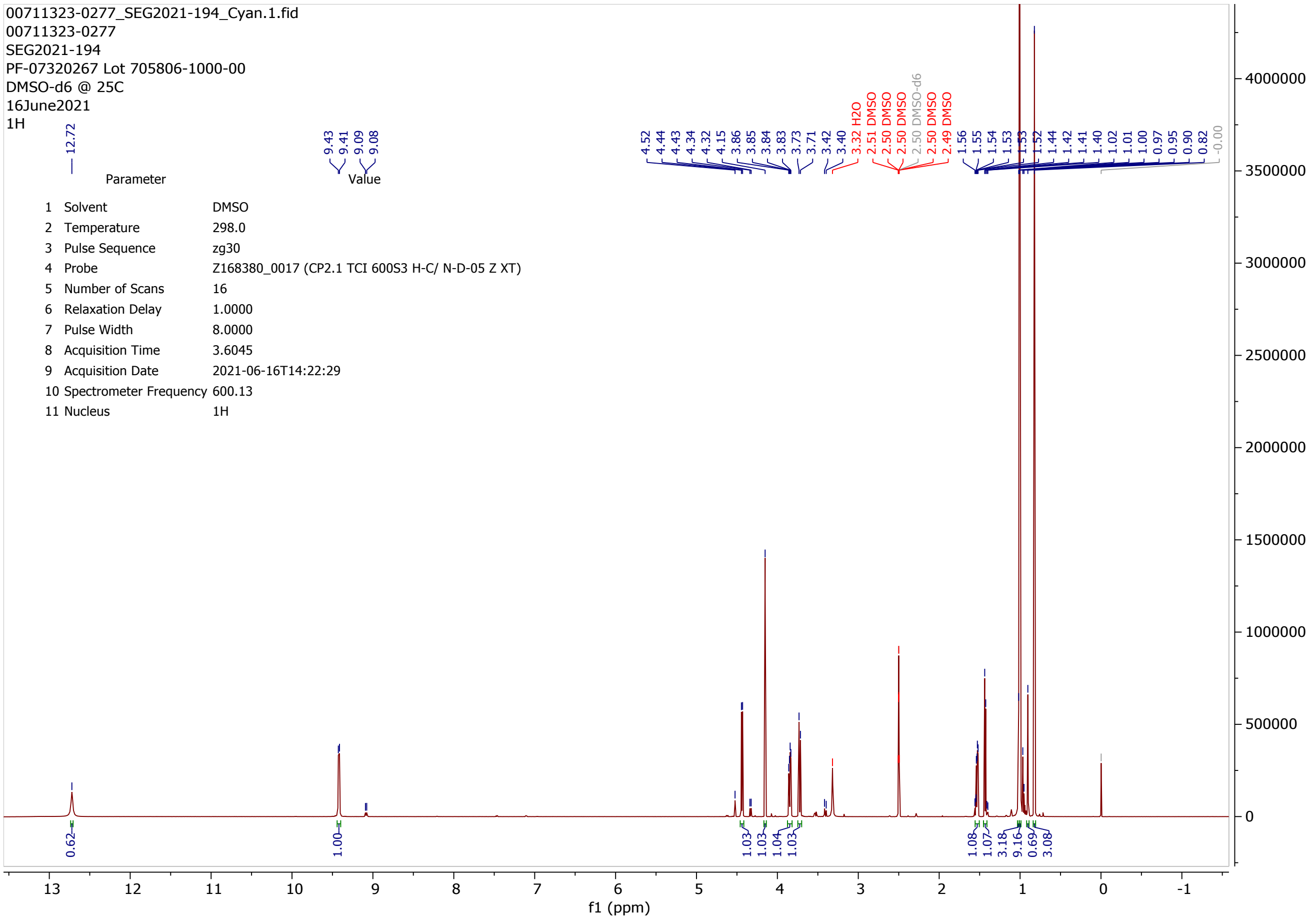


| | Parameter | Value |
|---|---|---|
| 1 | Solvent | DMSO |
| 2 | Temperature | 298.0 |
| 3 | Pulse Sequence | zg30 |
| 4 | Probe | Z168380_0017 (CP2.1 TCI 600S3 H-C/ N-D-05 Z XT) |
| 5 | Number of Scans | 16 |
| 6 | Relaxation Delay | 1.0000 |
| 7 | Pulse Width | 8.0000 |
| 8 | Acquisition Time | 3.6045 |
| 9 | Acquisition Date | 2021-06-16T14:22:29 |
| 10 | Spectrometer Frequency | 600.13 |
| 11 | Nucleus | 1H |

$^{13}$C NMR (150 MHz, DMSO-$d_6$, 298K): d 172.3, 167.6, 156.9 ($^2J_{CF}$ = 37 Hz), 115.8 ($^2J_{CF}$ = 288 Hz), 59.2, 58.1, 47.2, 34.7, 29.7, 26.7, 26.2, 25.7, 18.8, 12.1.

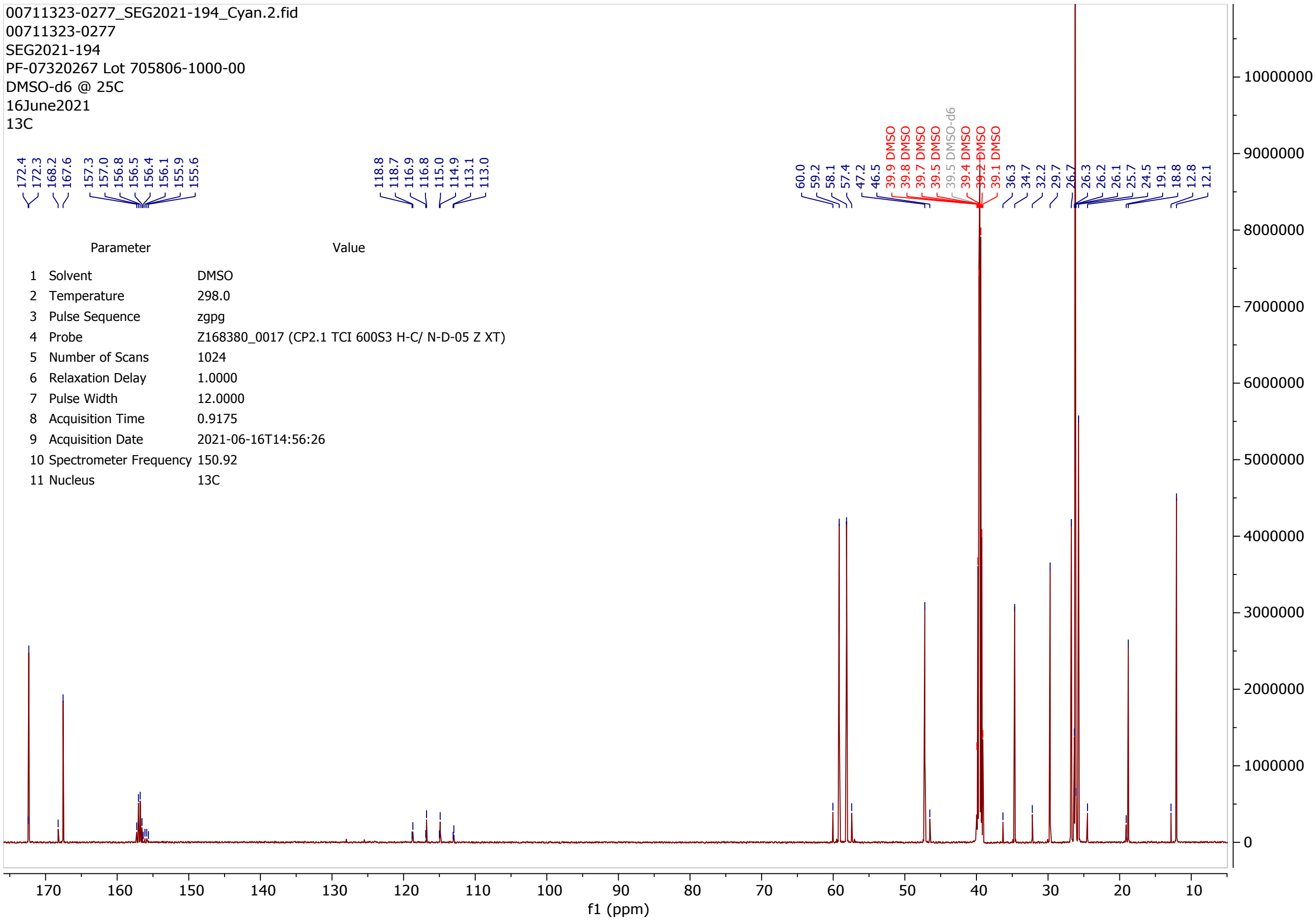


HRMS: (ESI$^+$) Calcd for $C_{16}H_{24}F_3N_2O_4^+$: 365.1610, Found: 365.1684 (mass deviation +0.5 ppm)

Step 3: Preparation of (1*R*,2*S*,5*S*)-N-((*S*)-1-amino-1-oxo-3-((*S*)-2-oxopyrrolidin-3-yl)propan-2-yl)-3-((*S*)-3,3-dimethyl-2-(2,2,2-trifluoroacetamido)butanoyl)-6,6-dimethyl-3-azabicyclo[3.1.0]hexane-2-carboxamide, compound **5**.

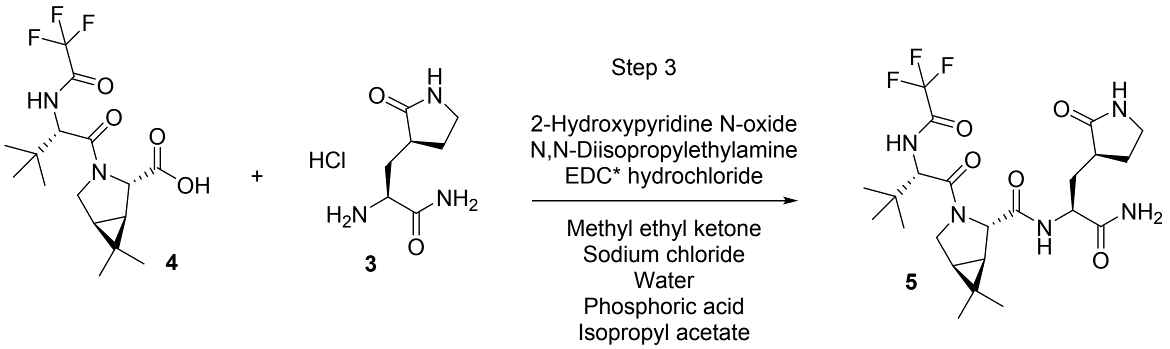


*EDC = 1-(3-Dimethylaminopropyl)-3-ethyl carbodiimide

In reactor A, (1*R*,2*S*,5*S*)-3-((*S*)-3,3-dimethyl-2-(2,2,2-trifluoroacetamido) butanoyl)-6,6-dimethyl-3-azabicyclo[3.1.0]hexane-2-carboxylic acid, compound **4** (1.0 kg, 2.74 mol, 1.0 equivalents) and methyl ethyl ketone (2.0 L, 2 L/kg of compound **4**) are combined and stirred at 25 °C. 2-Hydroxypyridine N-oxide (0.274 kg, 2.47 mol, 0.90 equivalents) and triethylamine (0.694 kg, 6.86 mol, 2.50 equivalents) are added, the resulting slurry is stirred for 30 minutes then warmed to 50 °C. In reactor B, (S)-2-amino-3-((S)-2-oxopyrrolidin-3-yl)propanamide hydrochloride, compound **3** (0.597 kg, 2.87 mol, 1.05 equivalents) and 1-(3-dimethylaminopropyl)-3-ethyl-carbodiimide hydrochloride (EDC, 0.682 kg, 3.56 mol, 1.30 equivalents) are combined with methyl ethyl ketone (3.0 L, 3 L/kg compound **4**), and the resulting slurry is stirred for 30 min then warmed to 50 °C. The solution from reactor A is then transferred into reactor B while maintaining the temperature at 50 °C in reactor B and stirring is continued for at least 6 hours. The reaction is sampled for completion (target of not more than 3% compound **4** remaining). If the reaction is not complete, the mixture is stirred for additional time. The reaction is quenched at 50 °C by the addition of aqueous NaCl (3.0 L of a 14 wt% brine solution, 3.0 L/kg of compound **4**) and stirring is maintained for 30 minutes. Stirring is stopped and the layers allowed to settle. The lower aqueous phase is removed, and the organic phase is washed with a second portion of aqueous NaCl (3.0 L of a 14 wt% brine solution), following the same protocol. The organic phase is cooled then concentrated by vacuum distillation at 0.3 bar while adding additional isopropyl acetate (18 L, 18 L/kg compound **4**) to maintain constant volume of ~6 L/kg, ending the distillation at 8 L/kg. A sample is analyzed for water content (Karl-Fischer) with a target of not more than 0.2 wt% water. The resulting organic solution of (1*R*,2*S*,5*S*)-N-((*S*)-1-amino-1-oxo-3-((*S*)-2-oxopyrrolidin-

3-yl)propan-2-yl)-3-((*S*)-3,3-dimethyl-2-(2,2,2-trifluoroacetamido)butanoyl)-6,6-dimethyl-3-azabicyclo[3.1.0]hexane-2-carboxamide (compound **5**) is used in Step 4 without further purification.

Step 4: Preparation of (1*R*,2*S*,5*S*)-N-{(1*S*)-1-cyano-2-[(3*S*)-2-oxopyrrolidin-3-yl]ethyl}-6,6-dimethyl-3-[3-methyl-N-(trifluoroacetyl)-L-valyl]-3-azabicyclo[3.1.0]hexane-2-carboxamide, methyl t-butyl ether solvate, compound **2**.

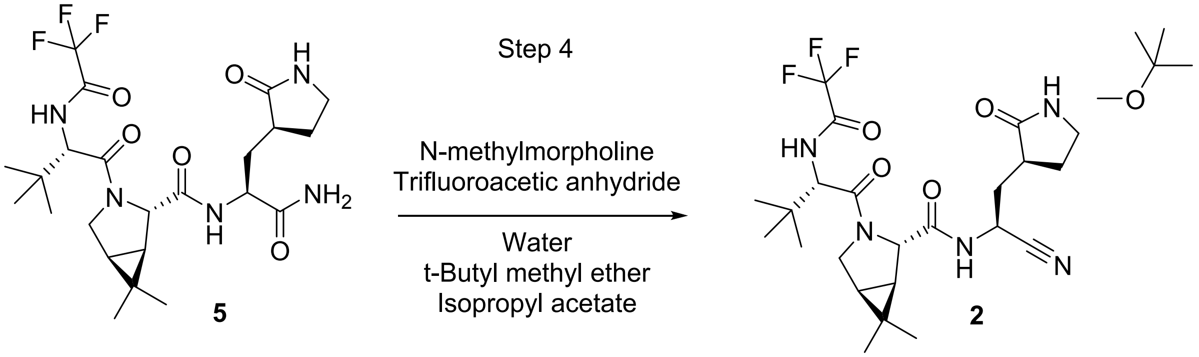


The isopropyl acetate solution of (1*R*,2*S*,5*S*)-N-((*S*)-1-amino-1-oxo-3-((*S*)-2-oxopyrrolidin-3-yl)propan-2-yl)-3-((*S*)-3,3-dimethyl-2-(2,2,2-trifluoroacetamido)butanoyl)-6,6-dimethyl-3-azabicyclo[3.1.0]hexane-2-carboxamide (compound **5**) prepared above in Step 4 (assumed quantitate conversion, 2.74 mol, 1.0 equivalents) is combined with N-methylmorpholine (1.11 kg, 10.4 mol, 4.0 equivalents) and stirred at 20 °C. Trifluoroacetic anhydride (1.15 kg, 5.20 mol, 2.0 equivalents) is charged over 60 minutes, maintaining the reaction temperature at not more than 25 °C. The resulting mixture is stirred for 1 hour. A sample is analyzed for reaction completion (not more than 0.5% of compound **5** remaining). If the reaction is not complete, maintain stirring for another 60 minutes, and charge additional trifluoroacetic anhydride if needed. The reaction is quenched by addition of water (3.0 L, 3.0 L/kg of compound **4** from previous step), stirring is maintained for 30 min, then stopped and the layers allowed to settle. The aqueous phase is removed, and the organic phase washed with a second 3.0 L portion of water. The organic phase is then concentrated by vacuum distillation (0.1 bar) to a volume of 3.5 L (3.5 L/kg of compound **4** from previous step). Isopropyl acetate (5.0 L, 5.0 L/kg of compound **4** from previous step) is added, and the solution is concentrated by vacuum distillation to a

volume of 3.5 L (3.5 L/kg of compound **4** from previous step). This solution is stirred at 50 °C, and methyl t-butyl ether (MTBE) is added over 60 minutes. If product crystallization does not occur during this addition, PF-07321332 MTBE solvate (compound **2**) seed (10 g, 1.0 wt% based on compound **4** from previous step) may be added. An additional portion of MTBE (6.0 L, 6.0 L/kg of compound IV from previous step) is added over 3 hours. This slurry is stirred at 50 °C for 1 hour, cooled to 20 °C at a rate of 0.1 °C /min, and stirred at 20 °C for 2 hours. Solids are collected by filtration, rinsed with 80:20 MTBE:iPrOAc solution (2.0 L/kg of compound **4** from previous step), and dried at not more than 50 °C. The product PF-07321332 MTBE solvate (**2**) was isolated as a white to off-white crystalline solid (1.17 kg, 73% yield over two steps).

Product characterization data was in agreement with that reported previously: Owen et al., Science 10.1126/science.abl4784 (2021). Characterization data is in the Supporting Information to that paper (pp. 15-16).

$^1$H NMR scan of compound **2** (DMSO-$d_6$):

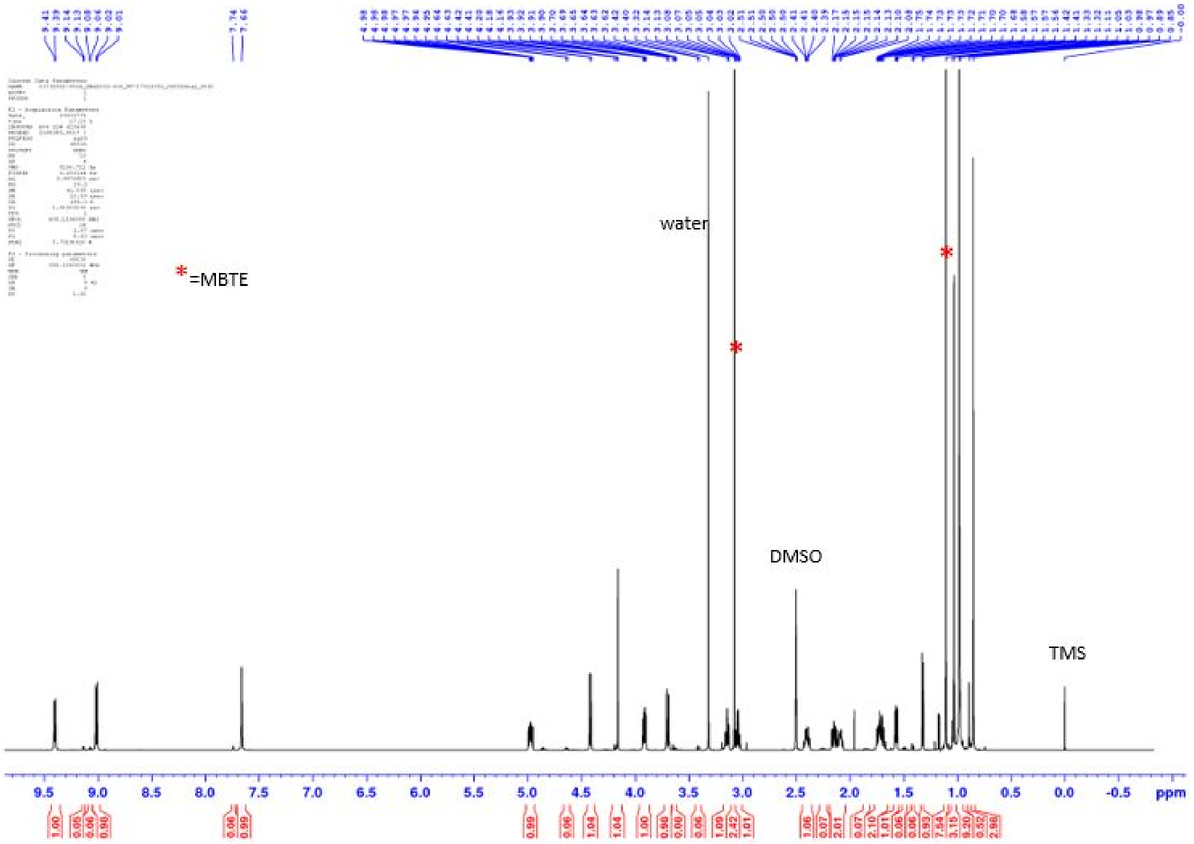


$^{13}C$ NMR scan of compound **2** (DMSO-$d_6$):

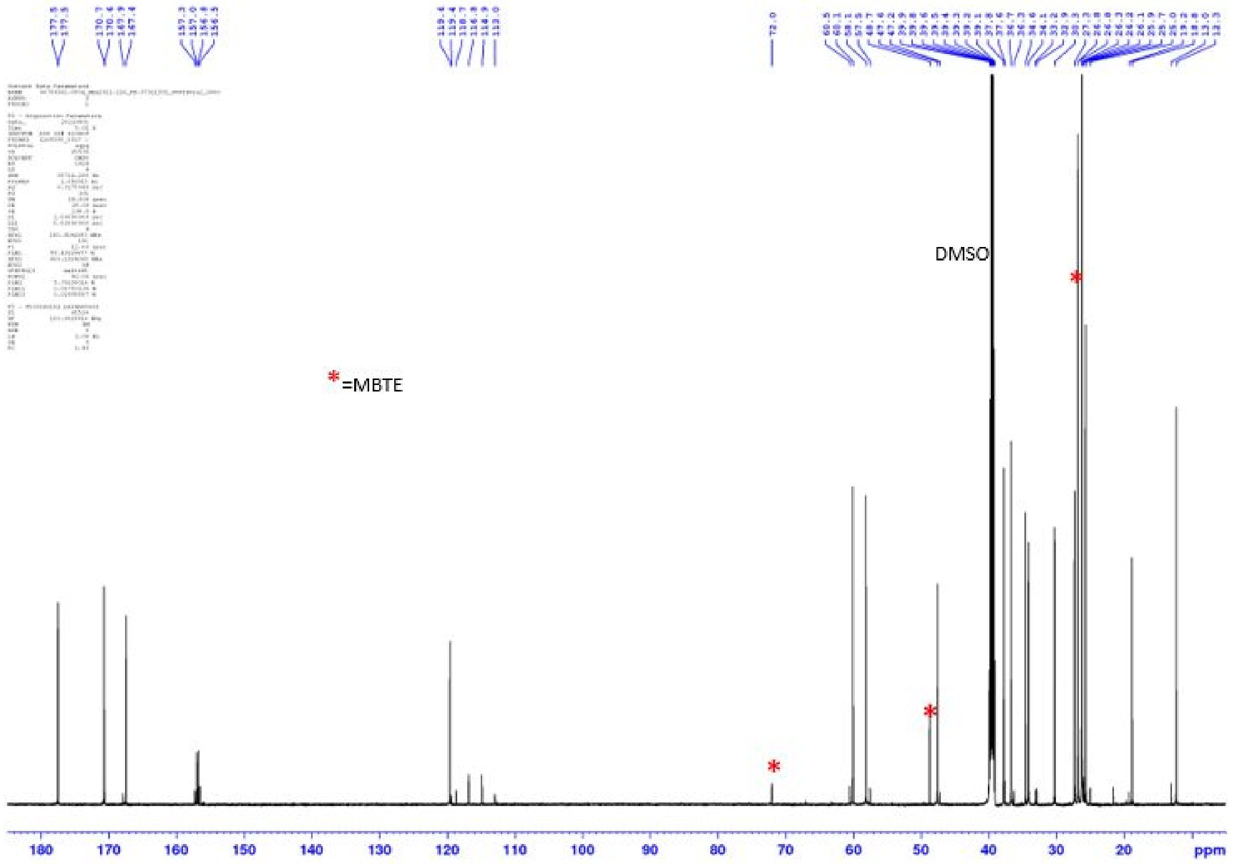


Step 5: Preparation of (1*R*,2*S*,5*S*)-N-{(1*S*)-1-cyano-2-[(3*S*)-2-oxopyrrolidin-3-yl]ethyl}-6,6-dimethyl-3-[3-methyl-N-(trifluoroacetyl)-L-valyl]-3-azabicyclo[3.1.0]hexane-2-carboxamide (nirmatrelvir), compound **1**.

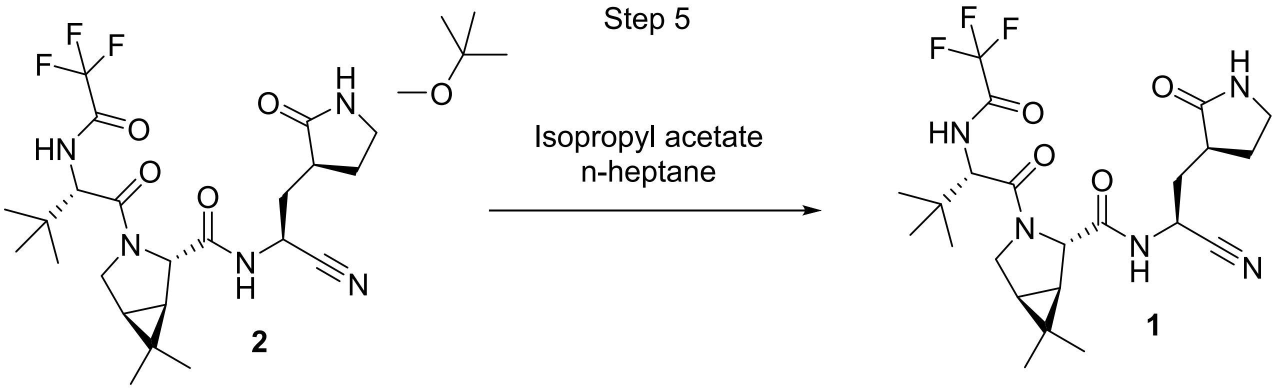

Compound **2** (50 g, 85.6 mmol) and isopropyl acetate (350 mL, 7 mL/g of compound **2**) are combined with stirring and warmed to 70 °C. A speck and fiber-free filtration may be performed at this stage if required. The solution temperature is adjusted to 65 °C and stirred for 15 minutes. Seed material is charged (compound **1**, 375 mg, 0.75 wt% based on compound **2**) and the mixture stirred for 30 minutes. N-heptane is charged over a period of 10 hours (600 mL, 12 mL/g of compound **2**), and the slurry is then cooled to 25 °C over 5 hours. Solids are collected by filtration, and the filter cake is washed with two 100 mL portions of 50:50 isopropyl acetate/n-heptane (200 mL total, 4 mL/g of compound **2**). The product is dried in a vacuum oven at NMT 50 °C, yielding 40.4 g of compound **1** (94% yield).

Product characterization data was in agreement with that reported previously: Owen et al., Science 10.1126/science.abl4784 (2021). Characterization data is in the Supporting Information to that paper (pp. 15-16).

$^1$H NMR scan of compound **1** (DMSO-$d_6$):

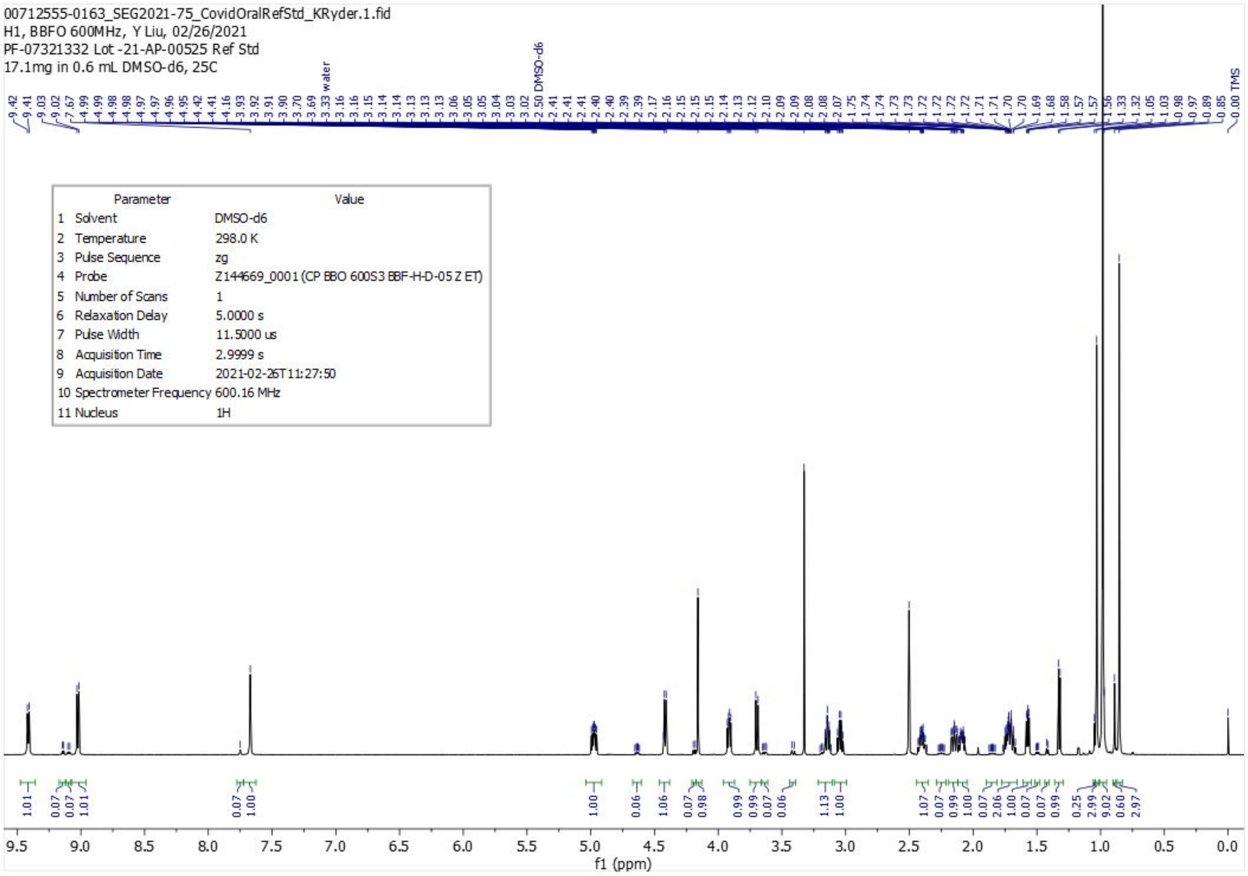

$^{13}$C NMR scan of compound **1** (DMSO-$d_6$):

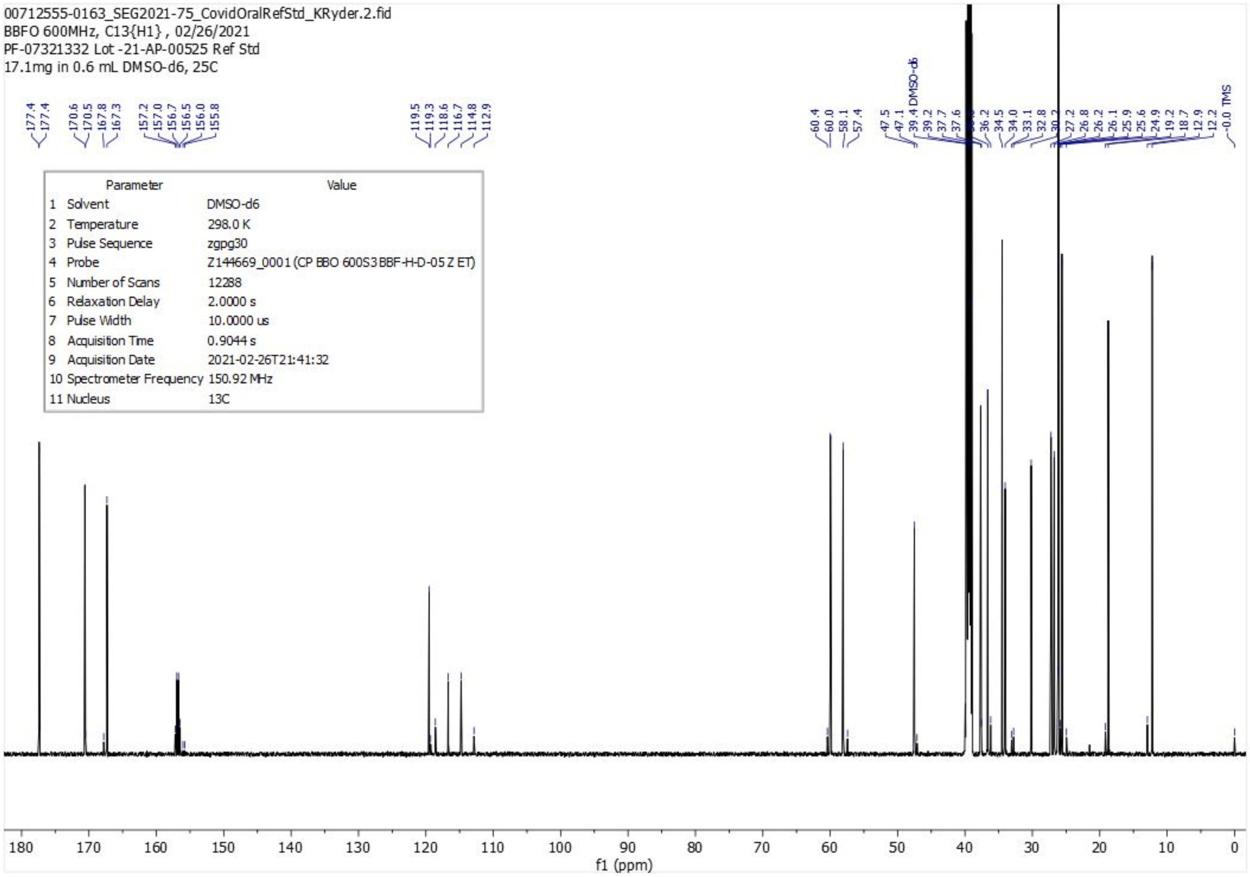


Preparation of methyl (1*R*,2*S*,5*S*)-6,6-dimethyl-3-azabicyclo[3.1.0]hexane-2-carboxylate, hydrochloride salt; compound **6**.

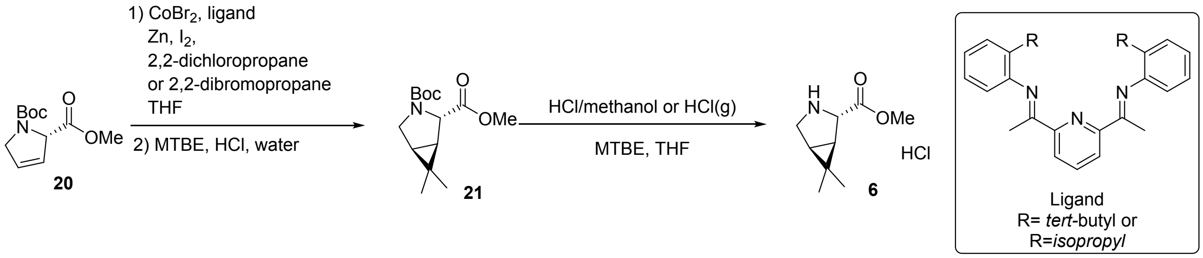


$CoBr_2$ (0.05-0.15 equiv), (1E,1'E)-1,1'-(pyridine-2,6-diyl)bis(N-(2-(*tert*-butyl)phenyl)ethan-1-imine) or (1E,1'E)-1,1'-(pyridine-2,6-diyl)bis(N-(2-isopropylphenyl)ethan-1-imine) (0.05-0.15 equiv, i.e. the ligand), and tetrahydrofuran (10 vol) were charged to a reactor. Zn (2.25-2.5 equiv.) was charged. $I_2$ (0.25 equiv.) in tetrahydrofuran (1-2 vol) was charged. A purple solution was obtained. 1-(*tert*-butyl) 2-

methyl (*S*)-2,5-dihydro-1H-pyrrole-1,2-dicarboxylate (1 equiv., compound **20**) was charged as a neat oil. 2,2-dichloropropane or 2,2-dibromopropane (1.5-2.0 equiv.) in tetrahydrofuran (1-3 vol) was slowly added and stirred until reaction completion (formation of compound **21**). The reaction mixture was filtered through Celite. Methyl tert-butyl ether (MTBE), HCl, and water were used during this operation. The organic phase was washed with HCl, water and dried with magnesium sulfate or sodium sulfate. The solution was concentrated to 1-2 volume. The resulting solution was treated with HCl (3 equiv.) in methanol or with tetrahydrofuran and HCl gas (3 equiv.). Upon reaction completion, methyl (1*R*,2*S*,5*S*)-6,6-dimethyl-3-azabicyclo[3.1.0]hexane-2-carboxylate, hydrochloride salt (compound **6**) was isolated from MTBE and THF as a solid. Typical yields are 50-80%. For example, 3.00 g of 1-(*tert*-butyl) 2-methyl (*S*)-2,5-dihydro-1H-pyrrole-1,2-dicarboxylate was converted to 1.98 g of methyl (1*R*,2*S*,5*S*)-6,6-dimethyl-3-azabicyclo[3.1.0]hexane-2-carboxylate, hydrochloride salt (80% yield). Product characterization data was consistent with that reported previously: Oruganti, S. et al. *Tetrahedron*, **2017**, *73*, 4285.

$^1$H NMR scan of compound **21** ($CDCl_3$):

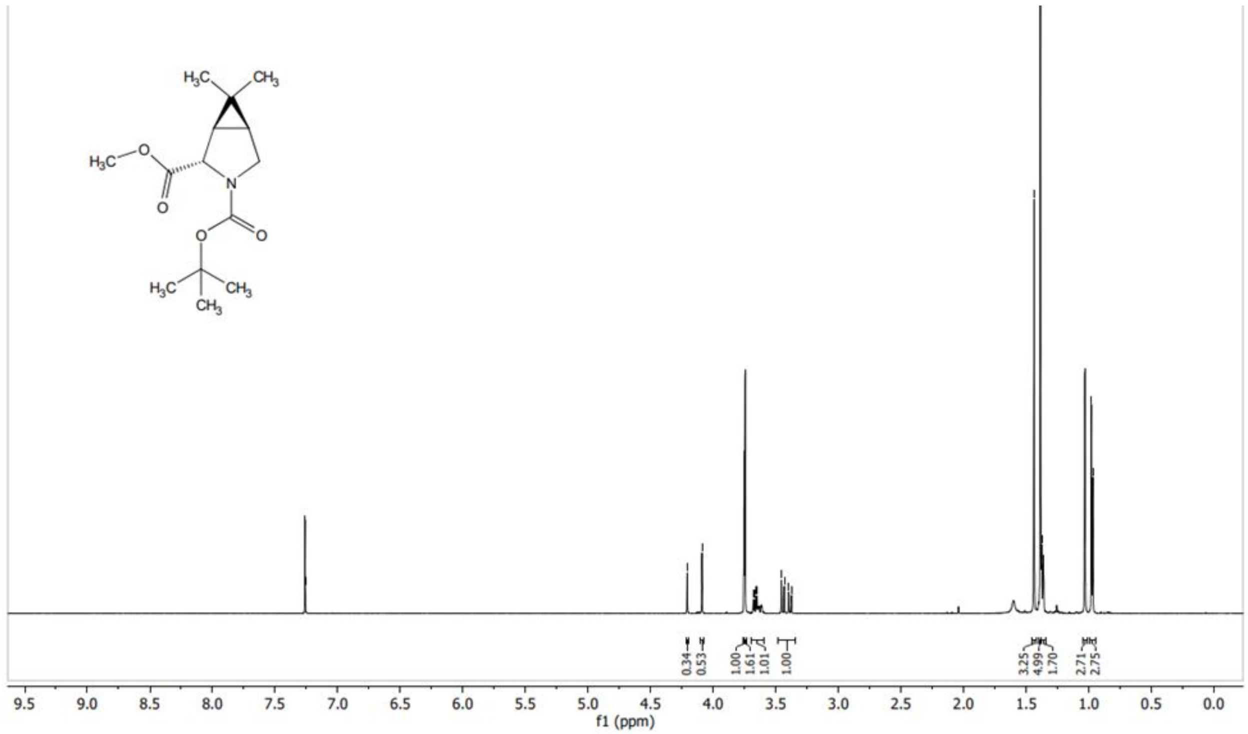

$^{13}C$ NMR scan of compound **21** ($CDCl_3$):

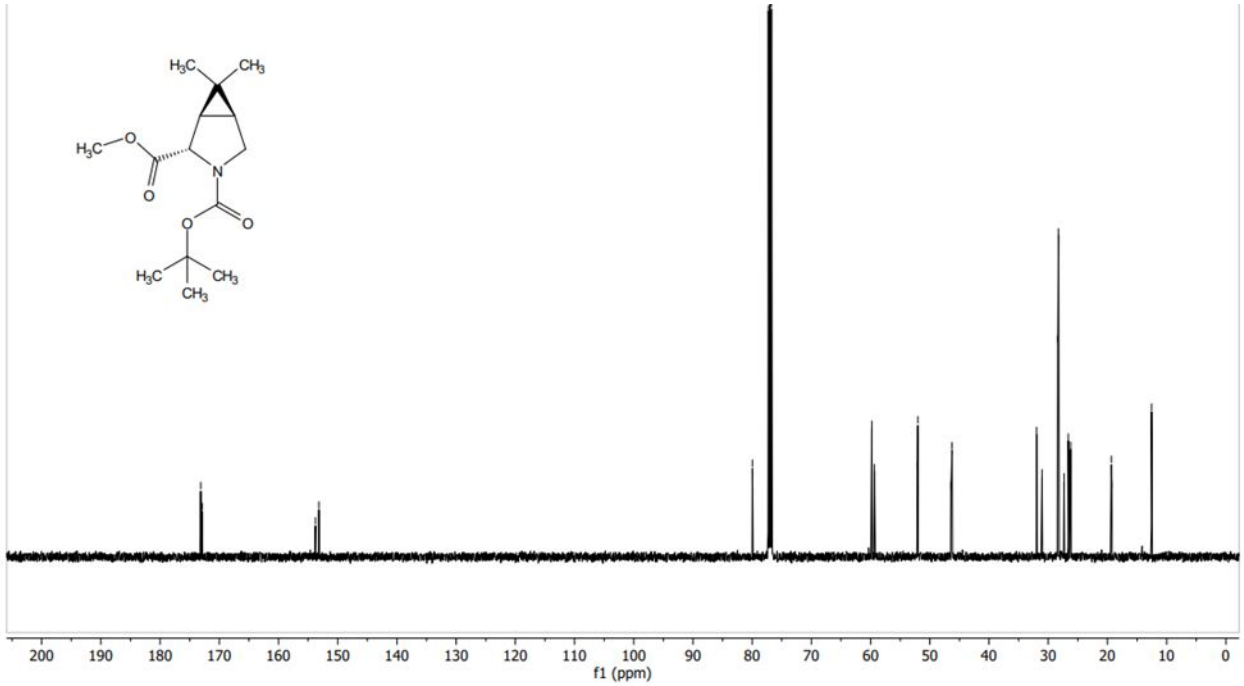


$^{1}H$ NMR scan of compound **6** (DMSO-$d_6$, 298K):

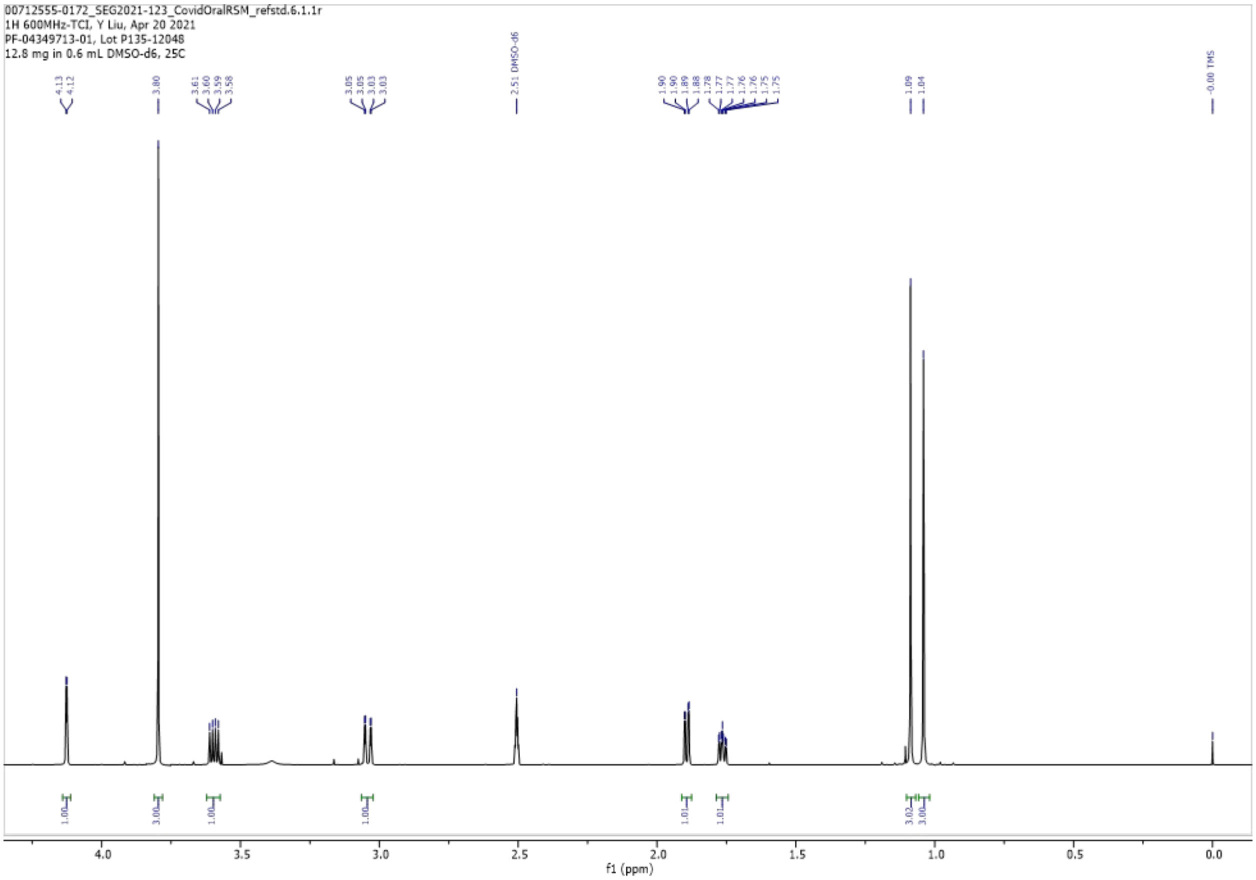


$^{13}C$ NMR spectrum of compound **6** (DMSO-$d_6$, 298 K):

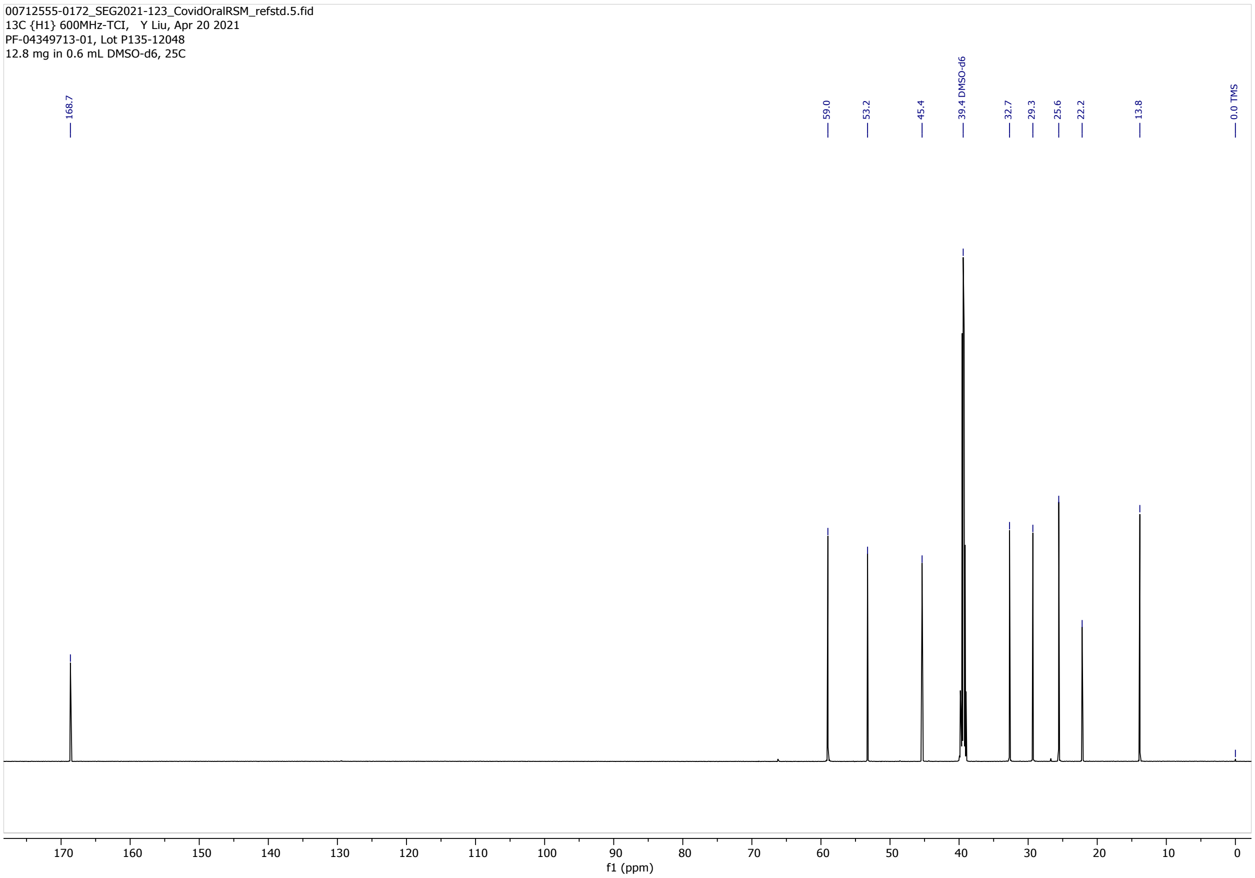


Preparation of (*S*)-2-amino-3-((*S*)-2-oxopyrrolidin-3-yl)-propanamide hydrochloride (compound **3**) via Route A.

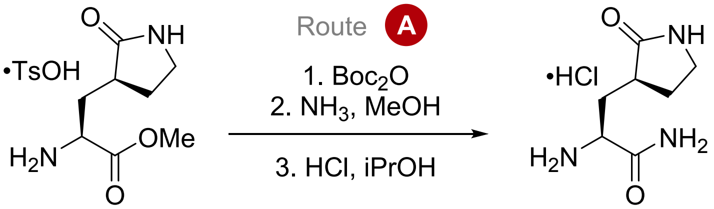


1. Methyl (*S*)-2-amino-3-((*S*)-2-oxopyrrolidin-3-yl) propanoate 4-methylbenzenesulfonic acid salt (compound **31**), triethylamine (1.2 equiv.) and di-tert-butyl-dicarbonate (1.2 equiv.) are combined in dichloromethane and stirred until reaction completion. Water is added, the phases are separated and the aqueous phase is extracted with dichloromethane. The combined organic layers are washed with aqueous sodium bicarbonate and water, then concentrated. Ethyl acetate is added and concentrated to a

volume of 1-2 L/kg of compound **31**. This mixture is stirred at 30 °C and methylcyclohexane is added slowly, and the resulting slurry is stirred at 10 to 20 °C for at least 2 hours. Solids are collected by filtration.

2. The solids are combined with methanol, cooled to 0 °C and treated with ammonia (10-20 equiv.), then warmed to 15 to 25 °C until reaction completion. Nitrogen gas is sparged through the solution, and the solvent is displaced with isopropanol.

3. The isopropanol solution is combined with 5 N aqueous HCl and stirred at 15 to 25 °C until reaction completion. The mixture is concentrated and displaced with additional isopropanol. Solids are collected by filtration and dried to provide compound **3**.

Preparation of (*S*)-2-amino-3-((*S*)-2-oxopyrrolidin-3-yl)-propanamide hydrochloride (compound **3**) via Route B.

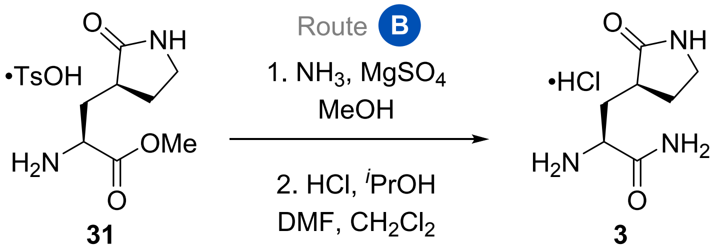


**31** **3**

1. Methyl (*S*)-2-amino-3-((*S*)-2-oxopyrrolidin-3-yl) propanoate 4-methylbenzenesulfonic acid salt (compound **31**), anhydrous magnesium sulfate (0.8 equiv.), aluminum oxide (0.5 equiv.) and 7 M methanolic ammonia (10 equiv.) are combined and stirred for 24 hours. The slurry is filtered, rinsing with methanol, and the resulting filtrate is concentrated and combined with dimethylformamide (5 L/kg of compound **31**).

2. This mixture is combined with 4 M HCl in isopropanol (1.8 kg/kg of compound **31**), seeded with compound **3**, and combined with dichloromethane (5 L/kg of compound **31**). Solids are collected, rinsed with dichloromethane, and dried to provide compound **3**.

$^1$H NMR spectrum of compound **3** (DMSO-$d_6$, 298 K):

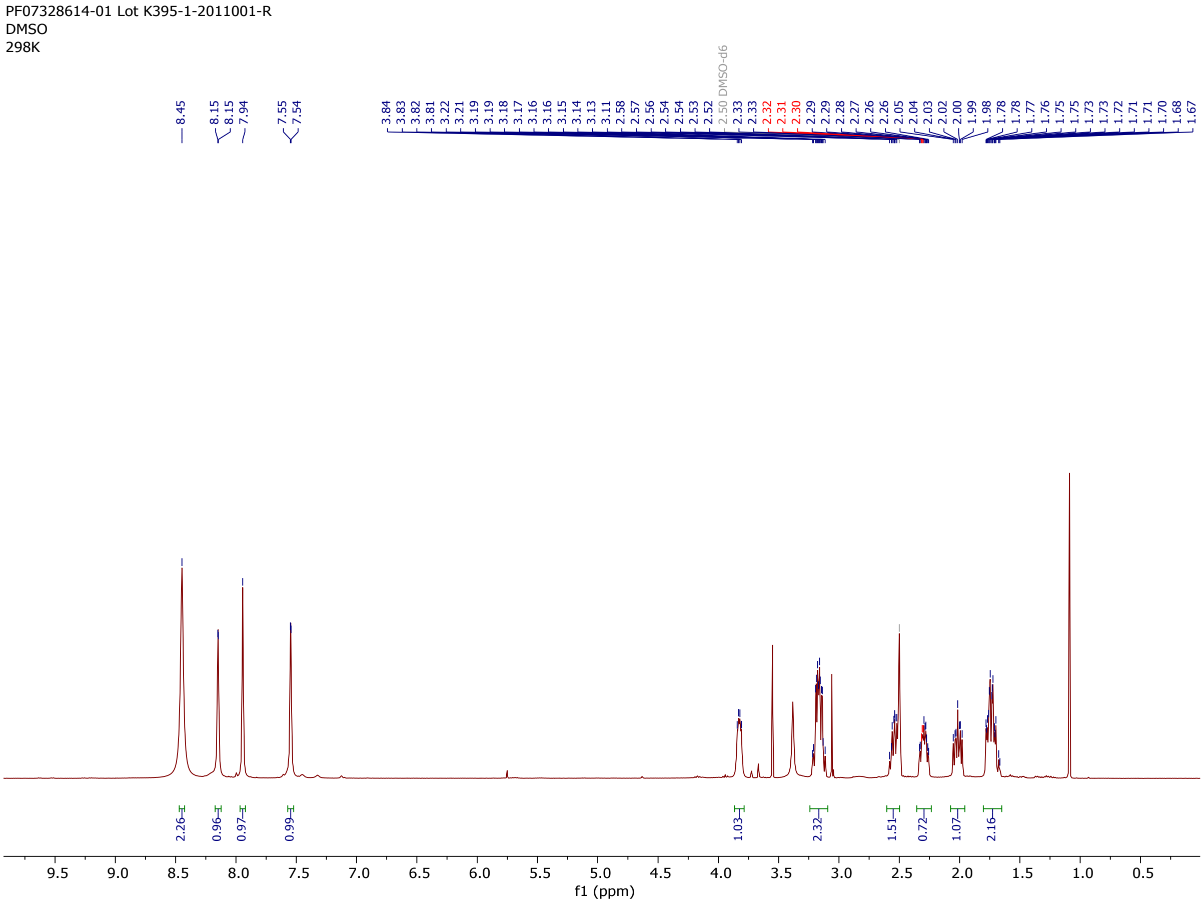


$^{13}$C NMR spectrum of compound **3** (DMSO-$d_6$, 298 K):

PF07328614-01 Lot K395-1-2011001-R
DMSO
298K

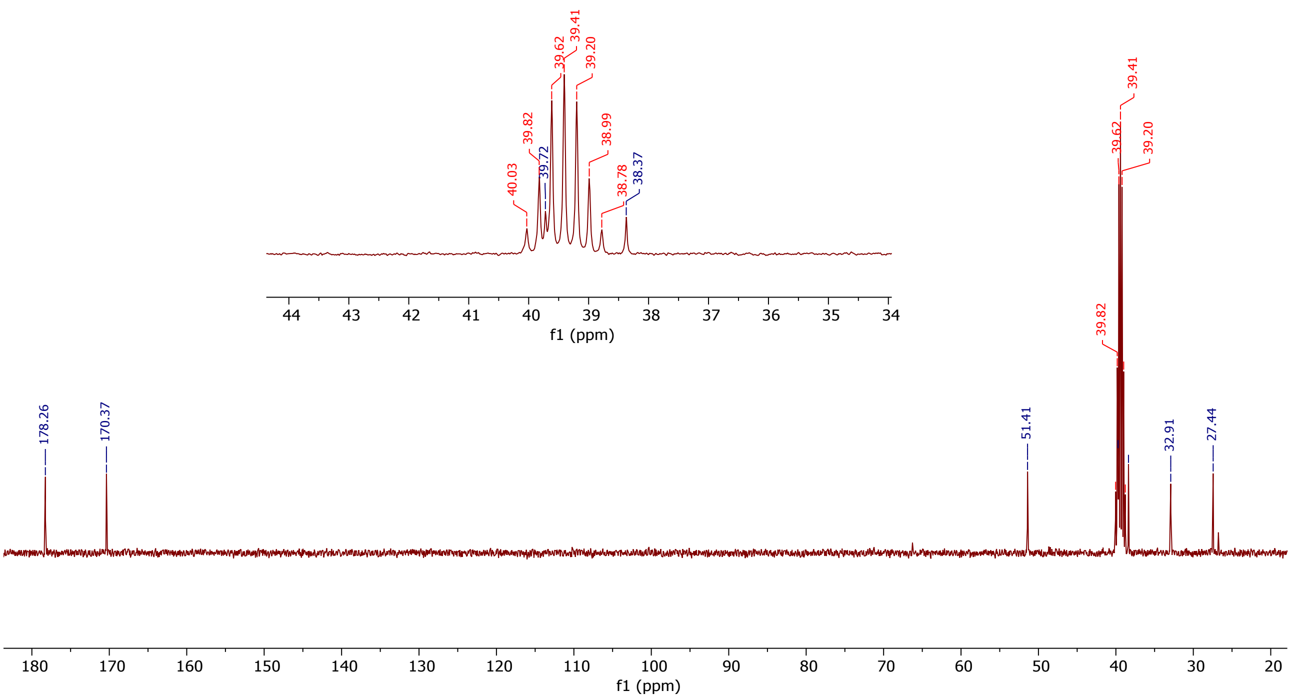

Supplement: Supplementary file 1 — oc3c00145_si_002.pdf [file oc3c00145_si_002.pdf]
